# Urosepsis by multidrug-resistant *Nakaseomyces glabratus* with non-functional Erg3 and Erg11—do collateral sensitivity and a unique mode of action make nitroxoline a viable UTI antifungal?

**DOI:** 10.1128/mbio.00588-26

**Published:** 2026-05-20

**Authors:** Hans Carolus, Vladislav Biriukov, Jolien Vreys, Celia Lobo Romero, Juan Paulo Herrera Avila, Rudy Vergauwen, Dimitrios Sofras, Jana Nysten, Giel Vanreppelen, Lore Vinken, Basil Britto Xavier, Toni Gabaldón, Katrien Lagrou, Reinout Naesens, Patrick Van Dijck

**Affiliations:** 1 Laboratory of Molecular Cell Biology, Department of Biology, KU Leuven 26657, Leuven, Belgium; 2 Département de Biochimie, de Microbiologie et de Bio-informatique, Faculté des Sciences et de Génie, Université Laval, Québec City, Québec, Canada; 3 Institut de Biologie Intégrative et des Systèmes (IBIS), Université Laval, Québec City, Québec, Canada; 4 PROTEO, Le regroupement québécois de recherche sur la fonction, l’ingénierie et les applications des protéines, Université Laval, Québec City, Québec, Canada; 5 Centre de Recherche en Infectiologie (CRI), Université Laval, Québec City, Québec, Canada; 6 Institute for Research in Biomedicine (IRB), Barcelona Institute of Science and Technology 518635, Barcelona, Spain; 7 Barcelona Supercomputing Centre (BSC-CNS) 132144 , Barcelona, Spain; 8 Department of Infectious Diseases, Ziekenhuis Aan de Stroom (ZAS) Antwerpen 682411, Antwerp, Belgium; 9 Department of Microbiology, Immunology and Transplantation, KU Leuven 418666 , Leuven, Belgium; 10 Department of Medical Microbiology and Infection Prevention, University of Groningen 3647, Groningen, the Netherlands; 11 Catalan Institution for Research and Advanced Studies (ICREA) 117370, Barcelona, Spain; 12 CIBER de Enfermedades Infecciosas, Instituto de Salud Carlos III, Madrid, Spain; 13 Department of Laboratory Medicine and National Reference Center for Mycosis, UZ Leuven 60182, Leuven, Belgium; 14 Department of Medical Microbiology and Infection Prevention, Ziekenhuis Aan de Stroom Antwerp 682411, Antwerp, Belgium; 15 KU Leuven One Health Institute, KU Leuven 26657, Leuven, Belgium; Yonsei University, Seoul, Republic of Korea

**Keywords:** *Nakaseomyces glabratus*, *Candida glabrata*, antifungal resistance, multidrug resistance, ERG3, ERG11, fitness trade-offs, collateral sensitivity, nitroxoline, urinary tract infection, amphotericin B resistance, urosepsis

## Abstract

The emergence of multidrug-resistant fungal pathogens from urinary tract infections (UTIs) poses a growing challenge in clinical settings. Here, we report a case of a complicated UTI caused by *Nakaseomyces glabratus* (*Candida glabrata*) that progressed to urosepsis, leading to the emergence of an isolate carrying simultaneous loss-of-function mutations in *ERG3* and *ERG11,* and abrogated ergosterol biosynthesis. Together with a missense mutation in *FUR1*—likely responsible for 5-fluorocytosine resistance—this constellation confers resistance to all viable UTI antifungals: azoles, amphotericin B, and flucytosine. Engineered *ERG3*Δ *+ ERG11*Δ strains recapitulated this multidrug resistance and revealed profound fitness costs that come with it, challenging the assumption that high-cost mutations are unlikely to persist during infection. Among fitness trade-offs, we detected collateral sensitivity to nitroxoline, a commonly used urinary tract antibiotic with potent antifungal activity and a unique mechanism of action. This study provides the first clinical evidence of an elusive mechanism of hyper-multidrug resistance in *N. glabratus* and highlights nitroxoline as a promising repurposing agent for treating multidrug-resistant fungal infections of the urinary tract.

**IMPORTANCE:**

Evolutionary theory states that fitness determines survival. In a drug-treatment environment, resistance increases fitness, but it often comes at a cost, such as slower growth or reduced stress tolerance. If these costs are too severe, they can undermine virulence, making resistance unlikely to persist. Our study challenges this assumption. We describe the first clinical case of *Nakaseomyces glabratus* evolving multidrug resistance through loss-of-function mutations in *ERG3* and *ERG11*, despite severe fitness trade-offs. This case suggests that certain infection niches, such as the urinary tract, can provide conditions where even highly impaired yet resistant strains persist under strong antifungal pressure. Importantly, we show that this extreme resistance induces collateral sensitivity to nitroxoline, a urinary tract infection antibiotic with potent antifungal activity and a unique mechanism of action. These findings open promising therapeutic avenues to counter multidrug-resistant fungal infections of the urinary tract.

## INTRODUCTION

Fungal infections form a major global health burden, causing around 6.5 million invasive infections and 3.8 million deaths annually. *Candida* species stand out among fungal pathogens due to their significant morbidity and mortality rates and increasing resistance to the limited arsenal of antifungal drugs. They account for an estimated 1,565,000 cases of bloodstream infections and invasive candidiasis, resulting in 995,000 associated deaths each year, reflecting a mortality rate of 63.6% (1). The spectrum of species causing candidemia has shifted from the traditionally drug-susceptible *Candida albicans* to non-albicans *Candida* (NAC) species, such as *Candida glabrata, Candida tropicalis, Candida parapsilosis,* and *Candida auris* (2). *C. glabrata*, which was recently renamed *Nakaseomyces glabratus* (3), causes 20 to 25% of invasive *Candida* infections in Western Europe and the USA (2) and is now one of the leading causes of candidemia in many healthcare settings, approaching the prevalence of *C. albicans*, which historically has been the dominant species (3).

In nosocomial settings, *N. glabratus* is implicated in approximately 21% of *Candida* urinary tract infections (UTIs) cases, second only to *C. albicans*, which causes approximately 49% of cases (4). While asymptomatic candiduria is often benign, symptomatic cases can manifest as cystitis, pyelonephritis, or other complications, particularly in high-risk patients. Fluconazole is the standard first-line treatment, but the effectiveness of this drug against *N. glabratus* is limited due to intrinsic resistance to azoles (4). Due to the poor urinary concentrations achieved by echinocandins, amphotericin B deoxycholate or flucytosine are recommended for UTIs of fluconazole-resistant NAC species (4–6).

Although amphotericin B has been a mainstay in antifungal therapy for over 70 years, resistance to this drug remains rare, which might be partially explained by the fitness trade-offs associated with the mechanisms of amphotericin B resistance (7–10). One elusive mechanism of hyper-resistance is the loss of function (LoF) of *ERG11,* which leads to the accumulation of toxic 14-methyl-ergosta-8,24(28)-diene-3,6-diol. The epistatic LoF of *ERG3* can mitigate this toxicity. Therefore, the combined LoF of both *ERG11* and *ERG3* has been identified as a putative mechanism of high amphotericin B resistance in several *in vitro* experimental evolution (7–9) and gene knock-out studies (11, 12). Nevertheless, this mechanism of resistance has been linked to severe fitness trade-offs (7, 9), which have been predicted to render the pathogen avirulent (8, 9). Thus far, LoF mutations in both *ERG3* and *ERG11* have only been documented in a clinical case of *C. tropicalis* (13). Here, we describe the first case of dual Erg3–Erg11 LoF in *N. glabratus,* from a patient who developed a complicated *N. glabratus* UTI progressing to urosepsis. This study investigates the genetic basis of resistance, its associated fitness trade-offs, and the potential for alternative therapeutics such as the UTI antibiotic nitroxoline, providing insights into antifungal resistance mechanisms and clinical management strategies.

## RESULTS

### Clinical case

**Fig 1:**
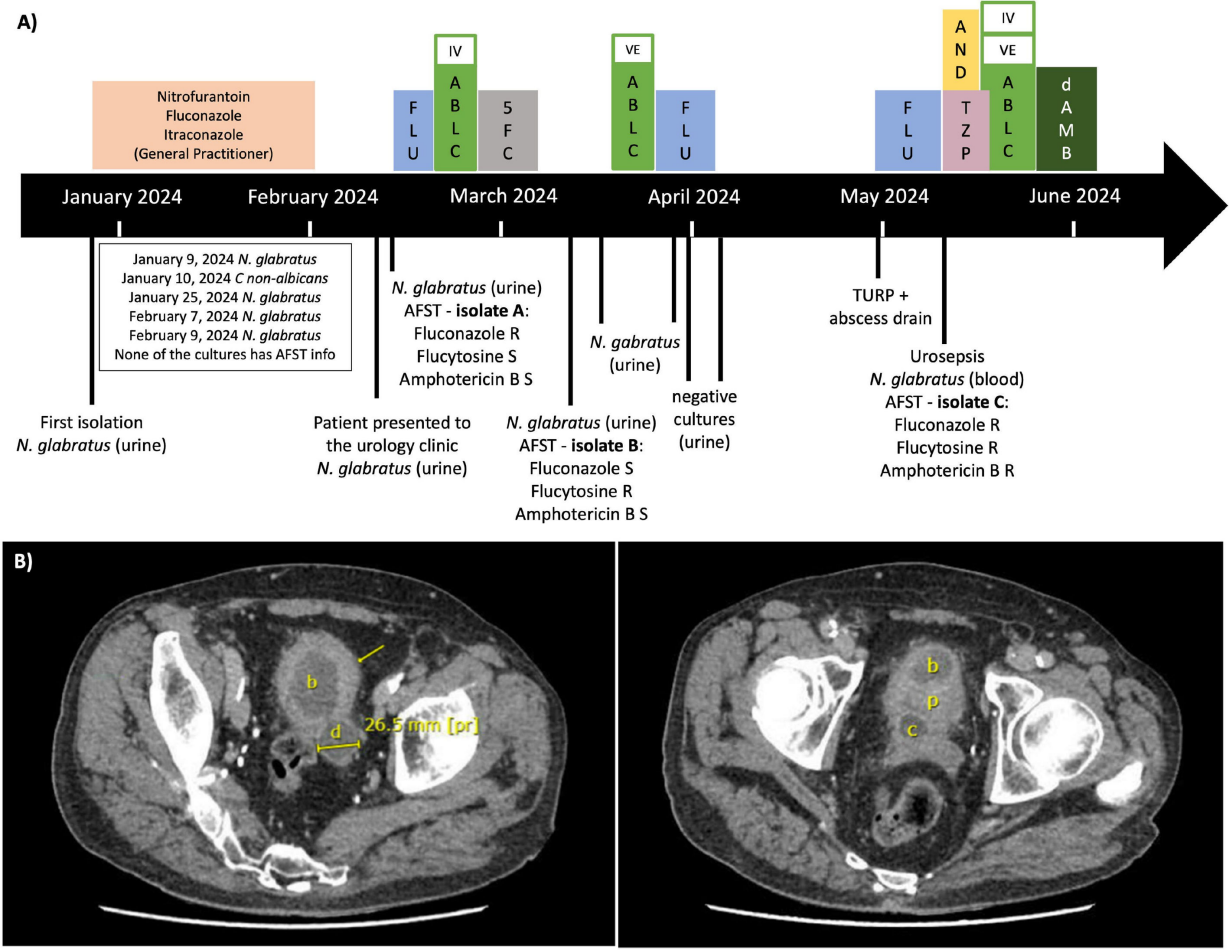
Timeline of isolation of *N. glabratus* and clinical manifestation. (**A**) The timeline shows the isolation of strains and antifungal susceptibility testing (AFST) data (below arrow) and treatment of the patient (above arrow). AFST details can be found in Table 1. FLU, fluconazole; ABLC, liposomal amphotericin B; VE, intravesical; IV, intravenously; 5FC, flucytosine; AND, anidulafungin; TZP, piperacillin-tazobactam; d-AMB, amphotericin B deoxycholate; TURP, transurethral resection of the prostate. AFST, antifungal susceptibility test. (**B**) Abdominal and pelvic CT scan in axial section with enhancement. Left image: bladder (b), bladder diverticulum (d), and thickened bladder wall (arrow). Right image: bladder (b), prostate (p), and cystic structure (c).

An 80-year-old male with a history of type 2 diabetes and stable ischemic cardiopathy presented to the ZAS urology clinic (Belgium) with several weeks of lower urinary tract symptoms and persistent isolation of *N. glabratus* in urine cultures (Fig. 1A). The patient had completed multiple courses of fluconazole without clinical improvement. Physical examination revealed no abnormalities. Rectal examination revealed a non-tender grade II prostate. Urinary tract ultrasound showed no significant abnormalities, with a structurally normal bladder and a postvoid residual urine volume of 20 mL. Prostate ultrasound revealed a homogeneous prostate with an approximate volume of 66 mL and overgrowth of the prostatic median lobe into the bladder (Fig. 1B).

**TABLE 1:** Clinical isolates with corresponding isolation date, site of infection, and AFST information, if obtained^*a*^

Isolate	Date and AFST method	Site	AFST		
			Drug	MIC (µg/mL)	Interpretation
A	14 February 2024, Sensititer YeastOne 10 (Thermo Fisher)	Urine	Anidulafungin	0.03	Susceptible
			Micafungin	0.03	Susceptible
			Caspofungin	0.5	Resistant
			5-Flucytosine	0.06	N.A.
			Posaconazole	>8	N.A.
			Voriconazole	4	N.A.
			Itraconazole	>16	N.A.
			Fluconazole	>128	Resistant
			Amphotericin B	1	Susceptible
B	11 March 2024, Sensititer YeastOne 10 (Thermo Fisher)	Urine	Anidulafungin	0.015	Susceptible
			Micafungin	0.015	Susceptible
			Caspofungin	0.12	Susceptible
			5-Flucytosine	64	N.A.
			Posaconazole	0.25	N.A.
			Voriconazole	0.25	N.A.
			Itraconazole	0.5	N.A.
			Fluconazole	4	Susceptible dose dependent
			Amphotericin B	1	Susceptible
C	10 May 2024, EUCAST	Blood	Anidulafungin	<0.008	Susceptible
			Micafungin	<0.008	Susceptible
			5-Flucytosine	>32	Resistant
			Posaconazole	>8	Resistant
			Voriconazole	>8	Resistant
			Itraconazole	>8	Resistant
			Fluconazole	>128	Resistant
			Amphotericin B	2	Resistant

^*a*^ Interpretation was performed according to the clinical breakpoints as determined by CLSI for Sensititer YeastOne 10 (14) and EUCAST for the EUCAST reference method (15). N.A. (not available) indicates no available clinical breakpoint.

Upon admission, the patient received fluconazole (400 mg daily, orally) for 5 days. The initial urine culture identified *N. glabratus* resistant to fluconazole but susceptible to flucytosine and amphotericin B (isolate A, Table 1). Consequently, fluconazole was discontinued, and amphotericin B lipid complex (670 mg daily, intravenously) was administered for 5 days. Treatment was subsequently switched to flucytosine (1,500 mg four times per day, orally) for 10 days. Despite 1 week of flucytosine therapy, the patient continued to experience dysuria and frequent urination. Cystoscopy revealed a bladder diverticulum with purulent drainage. A repeat urine culture identified *N. glabratus* resistant to flucytosine but susceptible to fluconazole and amphotericin B (isolate B, Table 1). Intravesical instillations of amphotericin B lipid complex (50 mg daily) were initiated, resulting in clinical improvement after 6 days. Fluconazole (400 mg daily, orally) was reintroduced for 8 days, leading to negative urine cultures. Unfortunately, symptoms recurred. A triple-phase abdomen and pelvis computed tomography (CT) scan revealed a thickened bladder wall, the previously known bladder diverticulum, and prostatic hypertrophy. Additionally, a 2 cm cystic structure was detected within the prostate (Fig. 1B). No evidence of fungus balls was found. The patient underwent a transurethral prostatectomy, during which purulent drainage and calcified tissue debris were collected from the cyst. Treatment with fluconazole (800 mg daily, orally) was initiated. Histological analysis revealed fungal structures in fibrinopurulent material from TURP and acute and chronic inflammation of the prostatic urethra with no evidence of malignancy (Fig. S1). Eleven days after the surgery, the patient presented to the emergency department with acute anuria caused by clot retention. A complete blood count showed leukocytosis with a white blood cell count of 14,600/μL (93% neutrophils), a decrease in hemoglobin (Hb) to 8.4 g/dL (baseline ~10 g/dL), and a normal to slightly elevated platelet count (432,000/μL). Serum creatinine increased significantly from the patient’s baseline of 1.3 mg/dL to 3.1 mg/dL, and C-reactive protein was markedly elevated at 160 mg/L. A single dose (1,250 mg) of amikacin was administered, and empirical therapy with piperacillin-tazobactam (4.5 g intravenously every 6 h) and anidulafungin (200 mg loading dose, followed by 100 mg daily for 3 days, intravenously) was initiated. Fluconazole was discontinued. The patient showed no improvement after 48 h. Blood and urine cultures were positive for *N. glabratus*, which was resistant to flucytosine, fluconazole, and amphotericin B (isolate C, Table 1). Treatment with ABLC was initiated via both intravenous (3 mg daily) and intravesical (50 mg daily) routes for 11 days. Meanwhile, the pharmaceutical team submitted an import request for amphotericin B deoxycholate, which was expected to achieve higher urine concentrations but was not available in Belgium. Subsequently, amphotericin B deoxycholate treatment (25 mg daily, intravenously) was initiated and maintained for 10 days. Follow-up in the urology clinic revealed significant clinical improvement, and subsequent urine cultures remained sterile 6 months post-treatment.

### Genome analysis and sequence data set mining

To investigate the mechanisms of acquired resistance to amphotericin B and flucytosine, we performed whole-genome sequencing and variant-calling analysis of the clinical strain (isolate C) using the ATCC2001 (CBS138) genome as a reference. To identify recently acquired variants that might explain the observed levels of resistance, we performed a phylogenomic analysis to determine the placement of this strain in the alignment of 420 *N. glabratus* isolates of Schikora-Tamarit and Gabaldón (16). Tree reconstruction revealed that the clinical isolate belongs to the *N. glabratus* clade 24 (16). A subset of the strains of this clade was previously also described as clade 7 (17). To differentiate clade-specific polymorphisms from potentially novel resistance-associated variants, we filtered out common variants present in 20% or more of the 51 strains within clade 24 (86,713 variants) from the variant calling results of the clinical strain. This yielded a list of 73 protein-altering (missense, frameshift, or nonsense) variants across 65 genes, listed in Table S1. Based on the functional annotation of the identified genes, we identified four mutations that can be linked to the resistance of this isolate, presented in Table 2.

**TABLE 2:** List of protein-altering variants in genes associated with antifungal drug resistance, found in the clinical isolate C^*a*^

Gene	Ortholog	Nucleotide change	Amino acid change	Variant type	BLOSUM62	SNAP2
CAGL0E04334g	*ERG11*	1543delC	L515fs	Frameshift	NA^*b*^	NA
CAGL0F01793g	*ERG3*	taC/taA	Y237*	Nonsense	NA	NA
CAGL0H09064g	*FUR1*	Ca/cAa	P138Q	Missense	−3	80
CAGL0L00671g	*FCY21*	aaG/aaT	K258N	Missense	0	6

^*a*^ Given are gene IDs in *N. glabratus* reference genome (ATCC2001/CBS138), gene aliases or orthologous gene names in *Saccharomyces cerevisiae*, nucleotide change indicating either specific variation in affected codons (reference allele/mutated allele) for single nucleotide polymorphisms (SNPs) or the nucleotide position in CDS for insertions (ins) or deletions (del). Predicted impacts of missense mutations on protein conformation were evaluated with SNAP2 (18) and BLOSUM62 (19). A higher SNAP2 score suggests greater impacts on protein structure and function. BLOSUM62 scores <0 indicate a low likelihood of an amino acid substitution to fixate by chance and can be used as a predictor for functional impact (20).

^*b*^ NA, not applicable.

The *ERG3* and *ERG11* genes encode essential components of the ergosterol biosynthesis pathway. *ERG11* encodes lanosterol 14α-demethylase, which catalyzes the demethylation of lanosterol, and *ERG3* encodes a C-5 sterol desaturase. The frameshift mutation in *ERG11* and the nonsense mutation in *ERG3* suggest the LoF of these genes. LoF mutations in *ERG11* result in the accumulation of sterols that have been suggested to be toxic to the cell and which are normally converted downstream to ergosterol. However, simultaneous LoF mutations in *ERG3* block this conversion and prevent the accumulation of this putative toxic sterol. The simultaneous LoF of *ERG3* and *ERG11* confers resistance to both amphotericin B and azoles, as ergosterol depletion reduces target binding, while also preventing the toxic effects of sterol intermediates, preserving viability (10, 12).

*FUR1 and FCY21* are linked to flucytosine antifungal activity and resistance. *FUR1* encodes uracil phosphoribosyltransferase, which catalyzes the conversion of 5-fluorouracil (5-FU) into the toxic metabolite 5-fluorouridine monophosphate (5-FUMP), a crucial step in 5FC activity. 5-FUMP inhibits both RNA and DNA synthesis, leading to cell death. Mutations in *FUR1* can prevent this conversion, rendering flucytosine ineffective (21). *FCY21*, a cytosine permease, facilitates the uptake of flucytosine into fungal cells. Mutations that impair *FCY21* activity can decrease flucytosine import, contributing to low-level resistance in *S. cerevisiae* (22). The high SNAP2 score and low BLOSUM62 score of the missense mutation in *FUR1* (Table 2) suggest a high functional impact and a rarely found amino acid substitution, respectively. The opposite is true for *FCY21* (Table 2)*,* suggesting this mutation might be of low impact and is likely to be the product of neutral genetic drift.

Because *FUR1* mutations have been investigated and associated with acquired flucytosine resistance to a great extent (21, 23), but the simultaneous LoF of *ERG3* and *ERG11* has not been reported in clinical isolates of *N. glabratus,* we further focused on the latter mechanism of resistance for further investigation.

We investigated whether variation in both *ERG3* and *ERG11* is common in *Candida* strains, by screening a data set of genomes of *C. albicans* (642 strains), *C. auris* (752 strains), *N. glabratus* (420 strains), *Candida orthopsilosis* (33 strains), *Candida parapsilosis* (51 strains), and *C. tropicalis* (89 strains) reported by Schikora-Tamarit and Gabaldón (16). Only protein-altering variants such as stop-gained, frameshift, splice-site in-frame insertions/deletions, and missense variants with a frequency lower than 0.2 (<20%) were considered in this analysis. For *N. glabratus* and *C. parapsilosis,* we did not identify cases of co-occurrence of *ERG3* and *ERG11* variants in the filtered clinical data set. In contrast, we identified isolates with co-altered genes for *C. auris* (1), *C. orthopsilosis* (1), *C. tropicalis* (6), and *C. albicans* (73), as shown in Table S2. In *C. albicans*, we found that the majority of strains carried often similar combinations of mutations, among which A351V and/or A353T mutation in the *ERG3* gene, along with E266D, V437I, S442F, and V488I variants in *ERG11*. Similar combinations were found enriched in the same clades. Among all analyzed *C. albicans* strains, only one strain demonstrated a rare combination of mutations in both genes: *ERG3* D14N, identified in one strain, and *ERG11* E336G, present in 3.27% of analyzed strains (21 strains). This suggests that most of such combinations might not represent recently emerged clinical adaptations but rather pre-existing variation present within specific clades that passed our 20% threshold at the species level. Additionally, all identified co-occurring mutations were missense variants, while the clinical isolate of this study shows a frame-shift and nonsense mutation in *ERG11* and *ERG3,* respectively (Table 2). Overall, the combination of high SNAP2 scores (>50) and low BLOSUM62 values (<1) for both *ERG11* and *ERG3* missense mutations was not detected. This suggests that high-impact variation in both *ERG3* and *ERG11* genes is extremely rare among *Candida* species. Consistently, we found no reports in the literature of combined LoF mutations in both *ERG3* and *ERG11* in clinical strains of *N. glabratus*.

### Validation of the mechanism of amphotericin B resistance

We further investigated the effects of the LoF of *ERG3* and *ERG11* by constructing single and double deletion mutants of both genes in the ATCC2001 (CBS138) background, followed by antifungal susceptibility testing and sterol analysis. The deletion of *ERG11* in the WT background could not be achieved in transformations under standard conditions but was successful upon plating transformed cells on agar supplemented with ergosterol and Tween 80 and incubating them anaerobically at 37°C, following recommendations from Geber et al. (11). Once transformed, *ERG11*Δ could be grown in normal growth conditions. The deletion of *ERG11* in the *ERG3*Δ background was achieved in standard transformation conditions. All attempts to transform the clinical strain were unsuccessful, potentially due to the poor growth, high stress sensitivity, and short life span of the isolate (see further).

**Fig 2:**
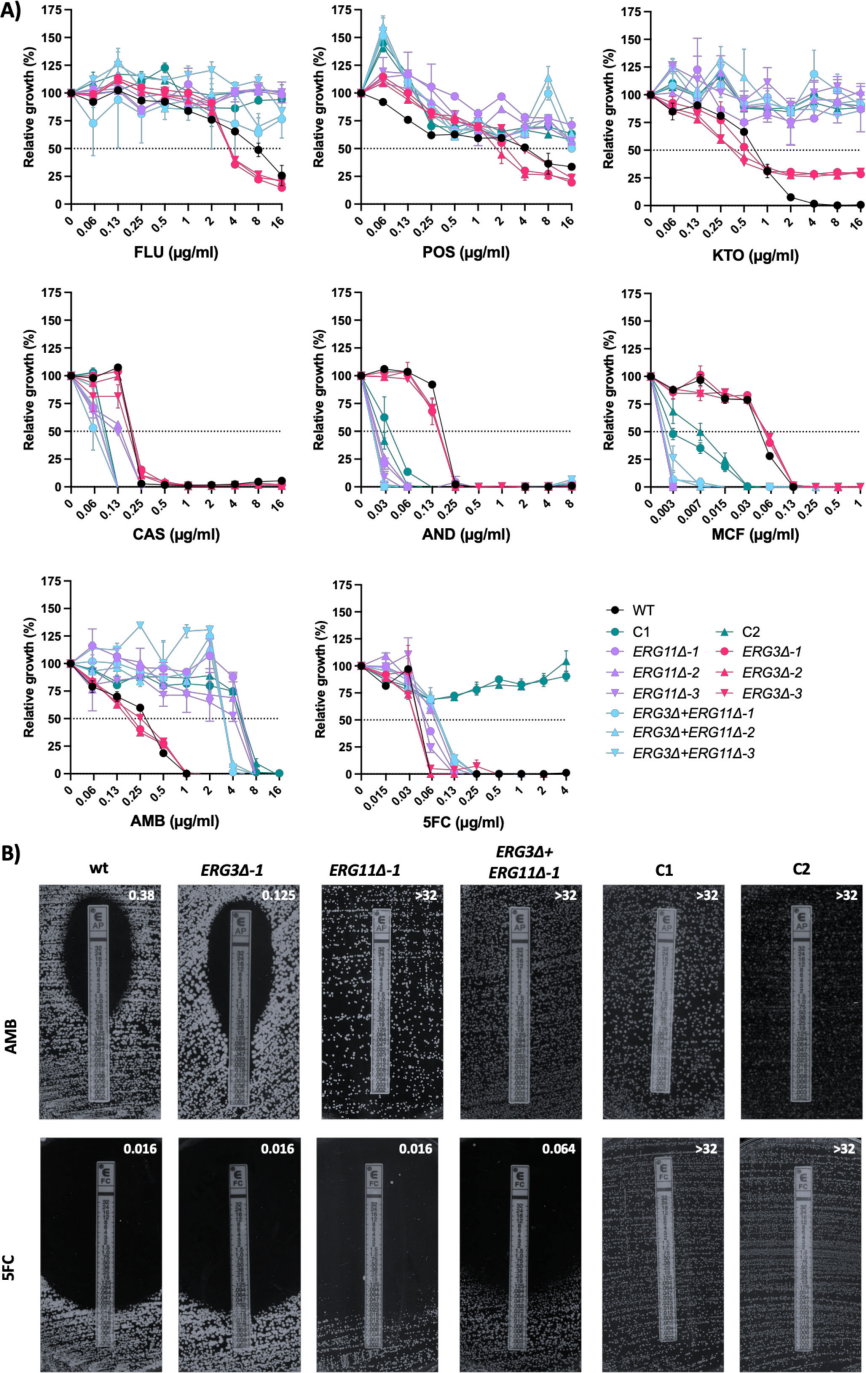
AFST of clinical and constructed strains. (**A**) BDA for FLU, posaconazole (POS), ketoconazole (KTO), caspofungin (CAS), AND, micafungin (MCF), amphotericin B (AMB), and 5FC for the WT *N. glabratus* (strain ATCC2001) and three biological replicates (independent transformants) per constructed genotype (i.e., *ERG3*Δ, *ERG11*Δ, and *ERG3*Δ *+ ERG11*Δ) in the WT background. Error bars indicate standard deviation (SD) based on two technical replicates. C1 and C2 are two independent single-colony isolates from clinical isolate C. (**B**) Pictures of ETEST (bioMérieux) after 72 h incubation at 37°C. The MIC value (in µg/mL) is read as the lowest concentration at which the border of the elliptical growth inhibition zone intercepted the strip and is indicated in the top-right corner of each image.

Broth dilution assays (BDA) showed that the LoF of *ERG11* led to amphotericin B and azole (fluconazole, posaconazole, and ketoconazole) resistance and resulted in collateral sensitivity (i.e., increased susceptibility to one drug class as a trade-off associated with resistance to another) to echinocandins (caspofungin, anidulafungin, and micafungin) (Fig. 2A). Simultaneous LoF of *ERG3* and *ERG11* yielded susceptibility patterns similar to the *ERG11* LoF strains. Interestingly, the *ERG3*Δ *+ ERG11*Δ strains showed a fourfold increase in flucytosine resistance, based on ETEST results (Fig. 2B). There is no clear link between altered membrane compositions and flucytosine resistance, but the effect is mild and does not explain the hyper-resistance to flucytosine in the clinical strain, which is likely the result of additional mutations in *FUR1, FCY21,* and/or other genes. *ERG3*Δ did not show altered susceptibility to amphotericin B, echinocandins, or flucytosine but did show slight changes in azole susceptibility: the MIC decreased for all azoles, while the higher supra-MIC growth (i.e., residual growth at concentrations above the MIC of 50% growth) was detected for ketoconazole.

**Fig 3:**
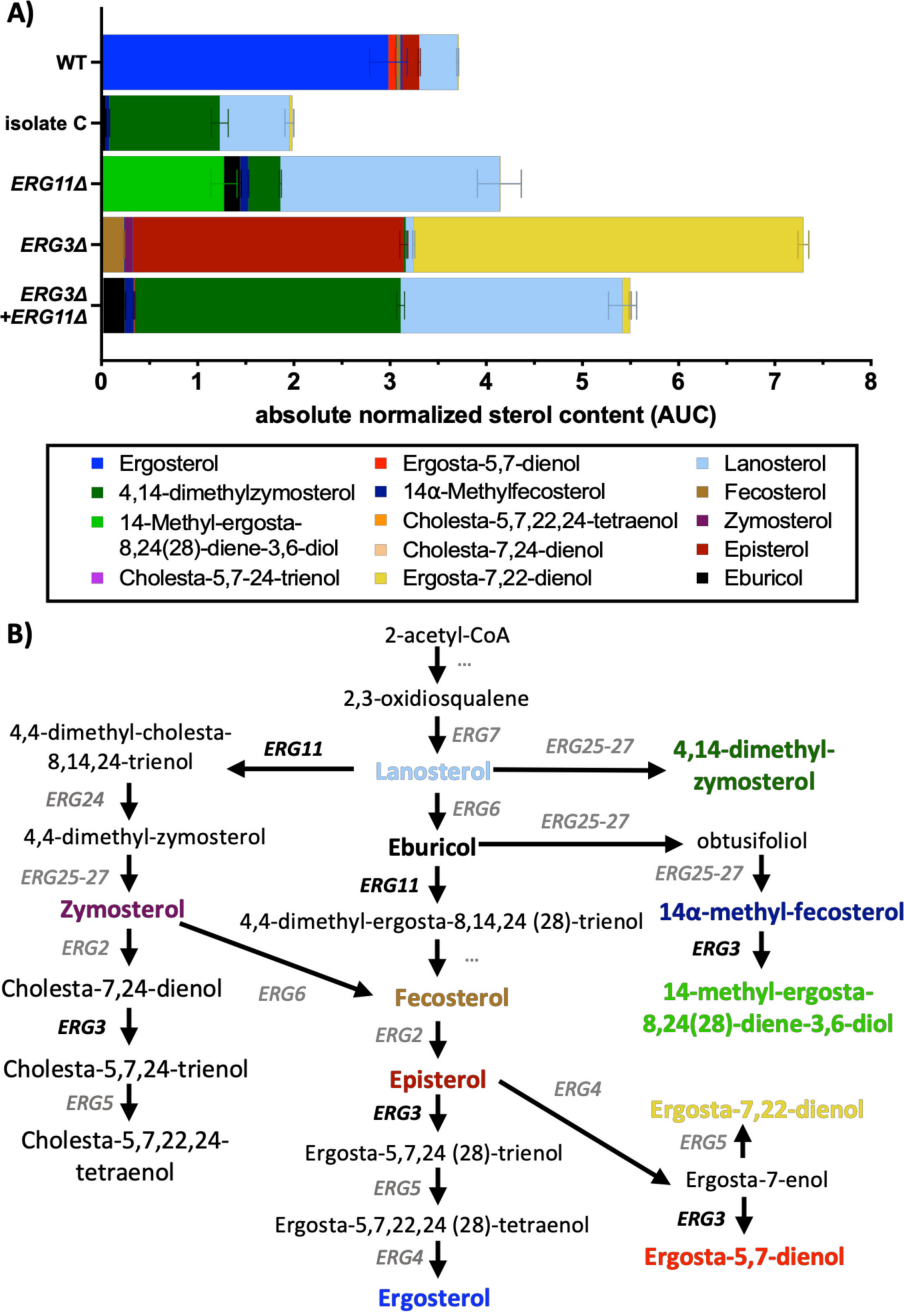
sterol quantification. (**A**) Sterol abundances are presented as GC-MS peak areas (AUC) normalized to the internal standard (5α-cholestane), providing a semi-quantitative comparison across strains. Cells are grown in drug-free growth conditions. Error bars indicate standard deviation from a minimum of two technical repeats (independent culture and GC-MS run) for one representative strain. (**B**) Simplified representation of the ergosterol biosynthesis pathway. Components and genes in bold are the main affected sterols and genes in our sequenced strains. The colors match the sterol compounds in the bar plots in panel A.

Next, we performed relative total sterol quantification on stationary growth-phase cells from all constructed and clinical strains, following Carolus et al. (9). The analysis shown in Fig. 3 indicates that ergosterol, which is dominant in the WT strain, is replaced mainly by 4,14-dimethyl-zymosterol and lanosterol in the *ERG3*Δ *+ ERG11*Δ strains and in the clinical strain (isolate C), confirming that Erg3 and Erg11 are non-functional in the clinical isolate. As expected, the *ERG11*Δ strains show the accumulation of 14-methyl-ergosta-8,24(28)-diene-3,6-diol, which is absent from *ERG3*Δ *+ ERG11*Δ or clinical strains. The deletion of *ERG3* leads to the domination of ergosta-7,22-dienol and episterol, which still might bind amphotericin B, as suggested by the susceptibility data presented in Fig. 2.

### Investigation of fitness trade-offs

**Fig 4:**
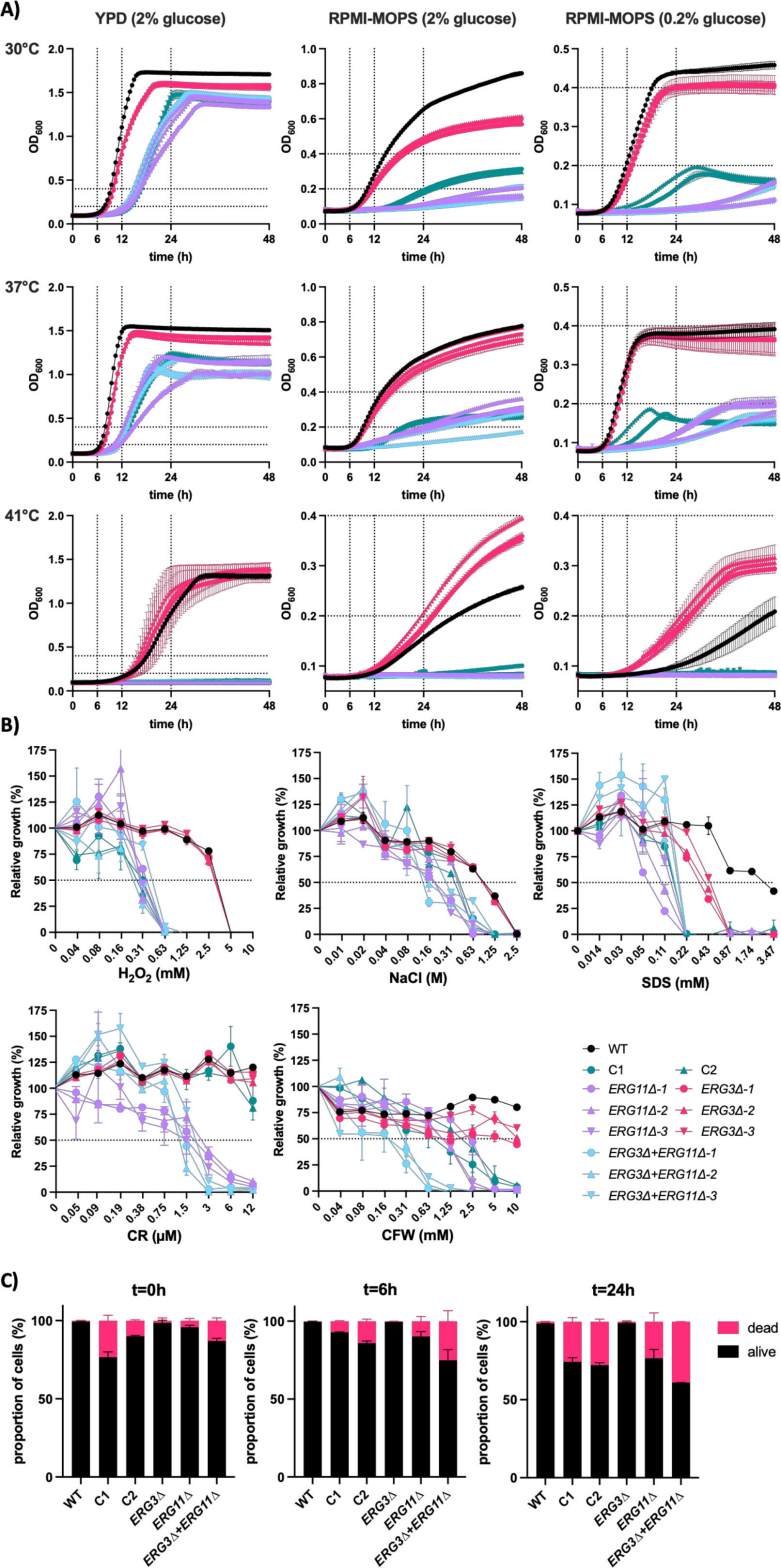
Fitness profiling of clinical and constructed strains. (**A**) Growth evaluation over 48 h at three different temperatures (30°C, 37°C, and 41°C) and in three different media (YPD 2% glucose, RPMI-MOPS 0.2% glucose, and RPMI-MOPS 2% glucose). Color and symbol legends for different strains are given in panel B. Error bars indicate standard deviation based on two technical repeats for each strain. (**B**) BDA depict the relative growth as a function of stressor concentration in RPMI-MOPS (pH 7, 2% glucose) after 48 h of incubation. The stress-inducing compounds used were calcofluor white (CFW), Congo red (CR), sodium dodecyl sulfate (SDS), sodium chloride (NaCl), and hydrogen peroxide (H_2_O_2_). Error bars represent the SD of two technical repeats per strain. (**C**) Relative proportion of live/dead cells in wild-type (WT), C1, C2, *ERG3*Δ-1, *ERG11*Δ-1, and *ERG3*Δ *+ ERG11*Δ-1 strains cultured in RPMI-MOPS medium (2% glucose) at 0 h, 6 h, and 24 h post-inoculation. Live/dead quantification was performed using fluorescent microscopy and differential staining as described in Materials and Methods and shown in Fig. S2. Error bars denote the standard deviation for two independent cultures per strain and time point.

Next, we performed growth, stress susceptibility, and survival analyses of all strains to estimate the burden of the LoF of *ERG3* and/or *ERG11* on cellular fitness. Growth curves shown in Fig. 4A demonstrate a strong reduction in growth rate and carrying capacity, and an increase in lag phase, due to the LoF of *ERG11* or *ERG3* and *ERG11,* in most growth conditions. The clinical strains seem to be slightly more adapted to low-temperature conditions (30°C) in physiological media (RPMI-MOPS 0.2% glucose and 2% glucose), compared to the *ERG3*Δ *+ ERG11*Δ and *ERG11*Δ strains. The LoF of *ERG3* does not significantly affect growth in most conditions, except in RPMI-MOPS 2% glucose, in which the growth rate and carrying capacity is significantly lower compared to the WT strain at 30°C but higher compared to the WT strain at 41°C in both 0.2% and 2% glucose RPMI-MOPS medium. Thus, the inactivation of Erg3 seems to impact thermotolerance in *N. glabratus*. The LoF of *ERG11,* on the other hand, renders any strain inviable at high febrile temperatures (41°C), as no growth was recorded for any *ERG11*Δ, *ERG3*Δ *+ ERG11*Δ, or clinical strain at 41°C in any of the growth conditions.

Stress susceptibility assays show that the LoF of *ERG11,* but not *ERG3*, significantly impacts fitness, as shown by the tolerance profiles for most stressors in Fig. 4B. Membrane stress tolerance (exerted by the detergent sodium dodecyl sulfate, SDS) is reduced to a lesser extent in *ERG3*Δ compared to *ERG11*Δ or *ERG3*Δ *+ ERG11*Δ strains, indicating that membranes enriched in episterol and ergosta-7,22-dienol are less stable than ergosterol-enriched membranes, but more stable or functional than those enriched with lanosterol, 4,14-dimethyl zymosterol and/or 14-methyl-ergosta-8,24(28)-diene-3,6-diol (Fig. 3). The tolerance to oxidative stress (hydrogen peroxide susceptibility) and osmotic stress (sodium chloride susceptibility) is reduced similarly for the *ERG11*Δ, *ERG3*Δ *+ ERG11*Δ, and clinical strains. Remarkably, the collateral sensitivity to the cell wall stressors Congo red and calcofluor white, seen in the *ERG11*Δ and *ERG3*Δ *+ ERG11*Δ strains, is not present or present to a lesser extent in the clinical strains, suggesting that additional mechanisms or the strain background play an important role in cell wall stress resistance.

During experimentation, we noted a discrepancy between estimated cell counts based on optical density (OD) in liquid culture and actual colony-forming units when plated on solid medium for the clinical strains and *ERG11*Δ mutants, suggesting a significant proportion of cell death. Therefore, we assessed relative cell viability over time in RPMI-MOPS 2% glucose liquid medium at 37°C, by live/dead staining and fluorescent microscopy. Figure 4C shows that a significant proportion of *ERG11*Δ, *ERG3*Δ *+ ERG11*Δ, and clinical strain (C1 and C2) cells are dead in these standard growth conditions, and that cell death increases over time.

In summary, the LoF of *ERG11* severely impacts fitness in terms of growth capacity, stress tolerance, and cell viability, while some of this reduced fitness is mitigated by the LoF of *ERG3* and/or background-specific differences.

### The therapeutic potential of nitroxoline

**Fig 5:**
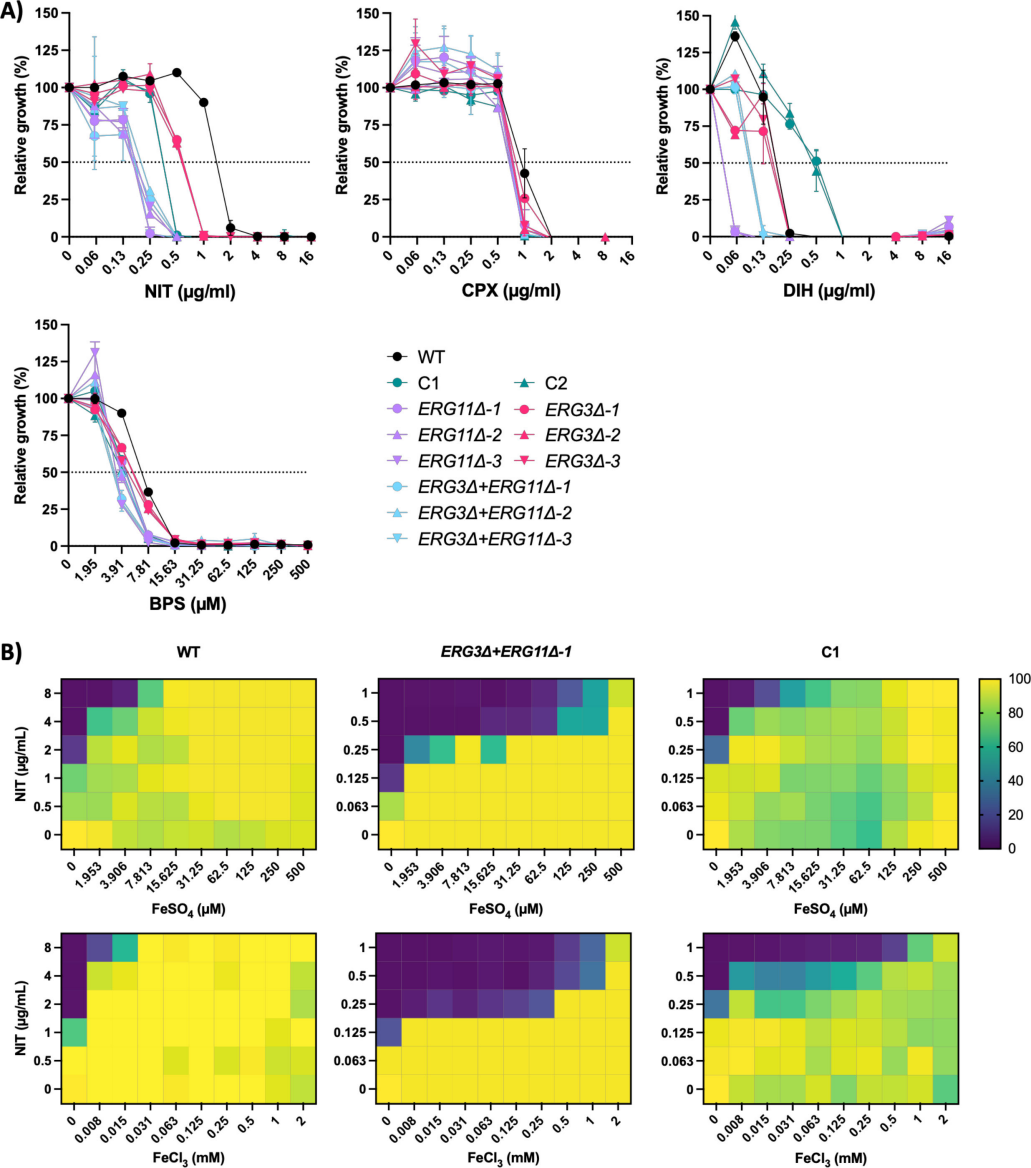
Evaluation of nitroxoline and iron chelation. (**A**) BDA depicted as the relative growth as a function of compound concentration in RPMI-MOPS (pH 7, 2% glucose) after 48 h of incubation at 37°C. Compounds used are nitroxoline (NIT), ciclopirox ethanolamine (CPX), diiodohydroxyquinoline (DIH), and BPS. Error bars represent the SD of two technical repeats per strain. (**B**) Iron rescue assays to evaluate the bioactivity of nitroxoline against the WT, *ERG3*Δ *+ ERG11*Δ, and clinical (C1) strain in the presence of FeSO_4_ and FeCl_3_. Nitroxoline and iron were each serially diluted twofold across a 96-well plate, similar to a checkerboard assay. Absorbance at 600 nm was measured after 48 h of incubation at 37°C, and the relative growth was plotted.

The poor urinary penetration of echinocandins (4, 5) and high resistance to amphotericin B, flucytosine, and azoles in this UTI case leave few therapeutic options. Recently, the antibiotic nitroxoline (8-hydroxy-5-nitroquinoline), which shows high urinary excretion, was reported to have antifungal activity against several *Candida* species, including *N. glabratus* (24, 25) and multidrug-resistant *C. auris* (26, 27). Therefore, we tested the susceptibility toward this compound and found that the clinical strain has a low MIC (0.5 µg/mL), while *ERG3*Δ*, ERG11*Δ, and *ERG3*Δ *+ ERG11*Δ exhibit collateral sensitivity to this drug, compared to the WT strain (Fig. 5A). The iron-chelating properties of the 8-hydroxyquinoline group, which is a privileged structure (28), are hypothesized to exert its antimicrobial activity, although other modes of action have been proposed (29, 30). To further evaluate whether the chelating properties indeed exert the antifungal and collateral-sensitive effect of nitroxoline on our tested strains, we evaluated the susceptibility toward the iron chelator bathophenanthroline disulfonic acid (BPS) and toward the drugs ciclopirox and diiodohydroxyquinoline. Ciclopirox is a topical antifungal of the hydroxypyridone class, which exerts its activity through iron chelation (31). Diiodohydroxyquinoline (8-hydroxy-5,7-diiodoquinoline or iodoquinol) is an antiprotozoal drug, which also exerts antifungal activity and is, like nitroxoline, an 8-hydroxyquinoline derivative (32). Figure 5A shows that the response to other iron-chelating agents (BPS and ciclopirox) or the 8-hydroxyquinoline analog diiodohydroxyquinoline is significantly different from that toward nitroxoline. The LoF of neither *ERG11* nor *ERG3* seems to affect the tolerance toward iron chelation, while the clinical strains show a higher MIC toward diiodohydroxyquinoline compared to the WT and constructed strains. This suggests that the antifungal activity of nitroxoline extends beyond the iron-chelating properties of its 8-hydroxyquinoline group. Differences in physicochemical properties between these compounds (e.g., lipophilicity, ionization state, and cellular uptake/permeability) and/or distinct intracellular metal-binding profiles may contribute to why nitroxoline elicits a collateral sensitivity phenotype that is not recapitulated by other chelators or by a related 8-hydroxyquinoline derivative.

To further test this hypothesis, we evaluated the inhibitory effect of nitroxoline in the presence of various concentrations of exogenous iron(II) (FeSO_4_) and iron(III) (FeCl_3_) in the WT, *ERG3*Δ *+ ERG11*Δ, and C1 strains. Figure 5B shows that, although iron supplementation can mitigate the inhibitory effect of nitroxoline in all conditions at a certain concentration, this rescue effect is less pronounced in the *ERG3*Δ *+ ERG11*Δ strain compared to the WT strain. For example, iron supplementation at low concentrations is insufficient to restore growth in nitroxoline concentrations at two- to fourfold the MIC (in iron-free conditions) in the *ERG3*Δ *+ ERG11*Δ strain, while in the WT strain, nitroxoline susceptibility is rescued even at the lowest concentration of iron. This indicates that the sensitivity of *ERG3*Δ *+ ERG11*Δ toward nitroxoline might be partially independent of iron chelation alone. A control experiment with the iron chelator BPS confirms this: Fig. S3 shows that the rescue effect of iron(II) (FeSO_4_) and iron(III) (FeCl_3_) to BPS is more similar for all strains.

**Fig 6:**
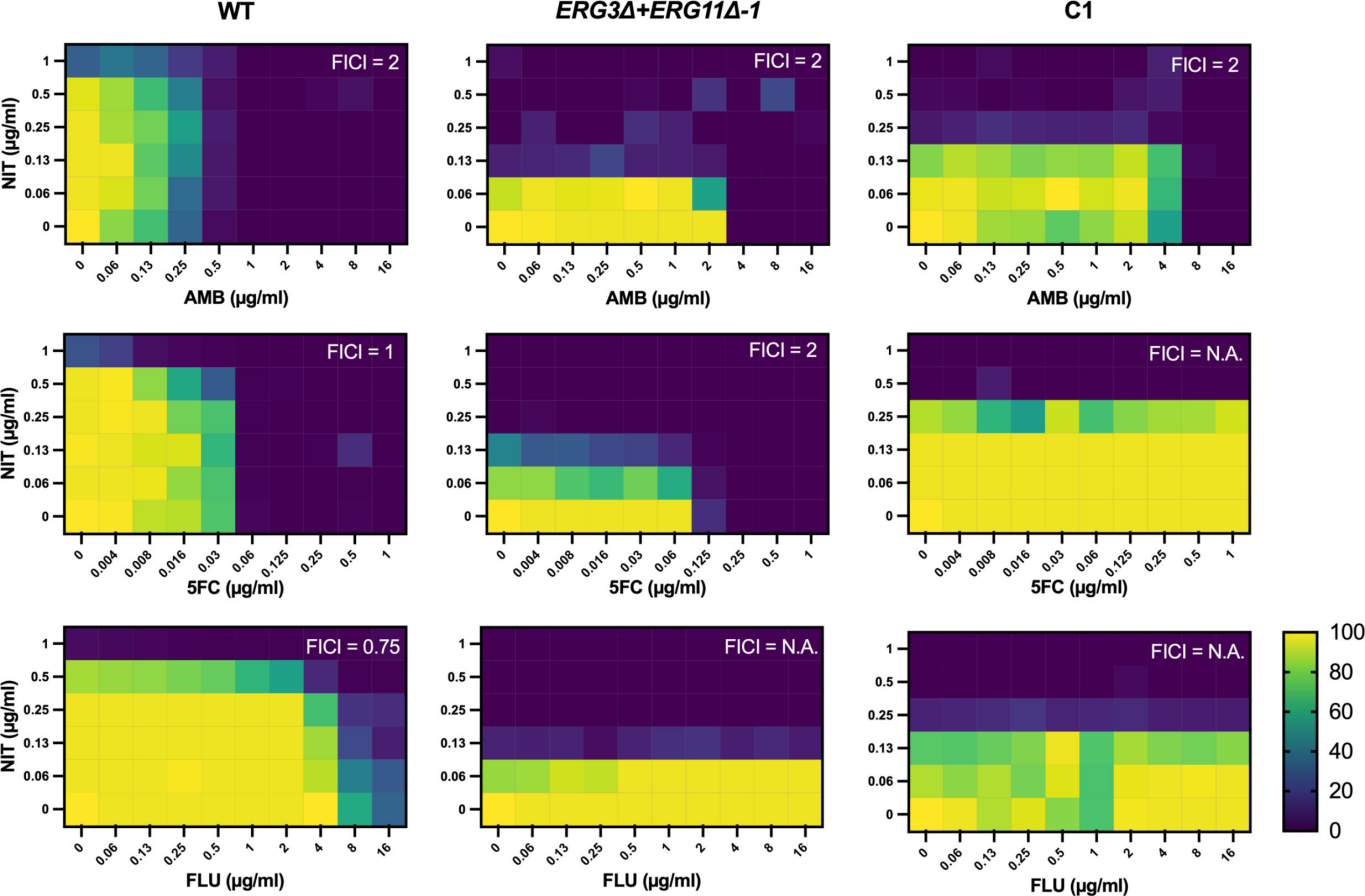
Checkerboard assays to evaluate drug interactions between NIT and AMB, 5FC, and FLU, respectively, for the WT, *ERG3*Δ *+ ERG11*Δ, and clinical (C1) strain. To evaluate drug interaction effects, the FICI was calculated and indicated in the upper right corner. $FICI=FIC_{drug1}+\;FIC_{drug2}=\frac{MIC_{drug1\;in\;drug2}}{MIC_{drug1}}+\frac{MIC_{drug2\;in\;drug1}}{MIC_{drug2}}$, with MIC_drug 1 in drug 2_ being the MIC of drug 1 in the presence of the highest concentration of drug 2 where growth is still visible (>50% compared to the control without drug). FICI ≤0.5 indicates synergy, 0.5 < FICI < 1 indicates an additive effect, 1 ≤ FICI < 4 indicates indifference, and a FICI >4 indicates antagonism (33). For checkerboard assays in which the MIC of one of the two drugs could not be determined within the tested range, no FICI could be calculated (indicated by N.A.).

Finally, we evaluated the interactive effects of nitroxoline and conventional antifungals—fluconazole, amphotericin B, and flucytosine—with checkerboard assays to determine whether this repurposed drug exhibits synergistic or antagonistic antifungal activity. Figure 6 shows that nitroxoline does not significantly decrease or increase the activity of any of the drugs toward the WT, *ERG3*Δ *+ ERG11*Δ, or clinical strain. The fluconazole-nitroxoline combination showed an additive effect with a fractional inhibitory concentration index (FICI) of 0.75, while all other drug combinations were indifferent or 1 ≤ FICI < 4 (33), or could not be calculated because the high resistance to the drug under investigation did not yield an MIC result within the tested range. Thus, nitroxoline could be used in combination with other UTI antifungal drugs without diminishing their effect, while in the case of azoles, it might increase the therapeutic outcome.

## DISCUSSION

This study uncovers a rare case of multidrug-resistant *N. glabratus* with dysfunctional Erg3 and Erg11 leading to severely impaired fitness that emerged under prolonged antifungal therapy in a complicated and persistent UTI that led to a breakthrough candidemia. LoF mutations in *ERG3* and *ERG11* resulted in resistance to amphotericin B, azoles, and decreased sensitivity to flucytosine, while mutations in *FUR1* and/or *FCY21* likely conferred high flucytosine resistance in the clinical isolate. Since polyenes, azoles, and flucytosine encompass most of the antifungal arsenal for treating UTIs, we explored the urinary tract antibiotic nitroxoline, which our results show to be a potent therapeutic alternative that may exert its antifungal activity via an unknown mechanism, unrelated to iron chelation. Drug interaction studies show that nitroxoline could be used in combination with other antifungals, with an additive effect between fluconazole and nitroxoline.

Prior work has shown that *ERG3* or *ERG11* defects individually can drive azole and/or amphotericin B resistance in *Candida* species (10, 34, 35), but the combined LoF of both genes is extremely rare, as shown by our *Candida* genome data set screening. After one *C. tropicalis* case from a Tunisian hospital reported by Eddouzi et al. (13), this is the second case in which both *ERG3* and *ERG11* LoF mutations confer azole and amphotericin B cross-resistance in a *Candida* strain of clinical origin. It is the first report of this mechanism of resistance in *N. glabratus*. In addition, the co-occurrence of hyper-resistance to flucytosine within this background is unique, and we hypothesize it is the result of additional mutations in *FUR1* and/or *FCY21,* which have both been implicated in flucytosine resistance (21–23).

The *ERG3*Δ *+ ERG11*Δ genotype has been investigated in several experimental evolution and gene knock-out studies (7–9, 11, 12). Our sterol analysis demonstrated that the LoF of *ERG3* and *ERG11* in *N. glabratus* leads to a 4,14-dimethyl-zymosterol and lanosterol-dominated sterol profile, which is similar to the effect observed in *C. albicans* (12) and *C. auris* (9), but different from the profile observed in *C. tropicalis,* which was dominated by 14α-methyl fecosterol, obtusifoliol, and eburicol (13). Another study in *N. glabratus* also shows 14α-methyl fecosterol and obtusifoliol, alongside lanosterol and 4,14-dimethyl-zymosterol (11). A potential explanation for this discrepancy might be that the sterol analyses in this study and the study of *C. albicans* (12) and *C. auris* (9) were performed on stationary phase cells (saturated cultures), while the analyses on *C. tropicalis* (13) and *N. glabratus* (11) were performed on cells in exponential growth phase (overnight culture) (13), which could result in the detection of short-lived intermediate sterols. Alternatively, significant differences could exist in sterol biosynthesis pathways between strains and species. The latter is supported by the observation that the *ERG3*Δ genotype leads to the accumulation of ergosta-5,7-dienol in this study, while this intermediate is absent in the same genotype in *C. auris,* grown and analyzed under the same conditions (9). Similarly, the LoF of *ERG3* has been reported to display resistance to azoles and cross-resistance to polyenes in *C. albicans* (8, 35) and *C. auris* (9, 36), while other studies and our results show no effect or even an increased susceptibility to either or both drug classes in the *ERG3*Δ background (11, 12). This, along with the fact that high-impact *ERG3* variation is rare (34), which is confirmed by our clinical data set screening, suggests that *ERG3* variation is no major driver of resistance in *N. glabratus*. The LoF of *ERG3* has been suggested to be an epistatic mechanism to compensate for the deleterious LoF of *ERG11,* rather than a stand-alone mechanism of polyene and/or azole resistance, which was also recently observed in acquired amphotericin B resistance in *C. auris,* where it was hypothesized to drive resistance *in vivo* (9). We do not see major differences in fitness between *ERG11*Δ and *ERG3*Δ *+ ERG11*Δ strains, suggesting that in *N. glabratus,* the LoF of *ERG11* and accumulation of 14-methyl-ergosta-8,24(28)-diene-3,6-diol is not necessarily toxic. Nevertheless, we were unable to knock out *ERG11* in standard transformation conditions in the WT background, while this was possible in the *ERG3*Δ background, suggesting that the LoF of *Erg3* does facilitate the LoF of *ERG11*. Beyond amphotericin B resistance, the epistatic LoF of Erg3 has been linked to the other types of resistance, such as echinocandin resistance and multidrug resistance (37). This complex role of *ERG3* in resistance to azoles, amphotericin B, and echinocandins highlights the importance of screening this gene in molecular diagnostics.

Because most types of sterol modulation that confer amphotericin B resistance impose significant fitness costs, it has been proposed that these changes reduce virulence and restrict the evolution of such mechanisms *in vivo*, making them less likely to occur in infections (8, 9). The simultaneous LoF of *ERG3* and *ERG11* has been reported to reduce the growth rate and stress tolerance significantly (8, 9, 11), to the extent that cells were assumed to be no longer able to maintain a bloodstream infection in an *in silico* infection model for *C. auris* (9). The fact that this genotype was isolated from a blood infection, and it showed hyper-resistance to flucytosine by an additional mechanism, challenges the notion that fitness trade-offs always undermine the evolution of resistance *in vivo*. This study shows that the imbalance of stronger drug resistance coupled with impaired growth, short lifespan, and high stress sensitivity as a cost does not hinder in-host adaptation. Although the selective pressure of antifungal treatment and/or the infection site (the prostate is a pharmacologic sanctuary site) may favor severely compromised mutants, the breakthrough candidemia of this isolate shows that these strains can persist and cause recalcitrant infections of other niches. It is important to note, though, that besides “isolate C,” no other clinical isolates were stored or could be investigated. Whether the *ERG3* and *ERG11* LoF mutations were acquired in the UTI or later, during candidemia, is therefore unknown. Consistent with a broader capacity for in-host adaptation, serial-isolate studies have documented rapid microevolution of *N. glabratus* during antifungal therapy, in which azole resistance and echinocandin resistance can arise alongside additional genomic changes over days to weeks (38–40). However, to our knowledge, the clinical emergence of the specific constellation described here—simultaneous *ERG3*/*ERG11* loss-of-function leading to pan-resistance to all viable UTI antifungals together with profound fitness impairment—has not previously been reported in *N. glabratus*.

Hardly any antifungal drugs can be used in fungal infections of the urinary tract in which resistance to polyenes, azoles, and flucytosine is acquired. Therefore, we investigated the *in vitro* potency of nitroxoline, an antibiotic with high urinary excretion and antifungal activity (24–27). All strains were susceptible to nitroxoline, and additionally, the *ERG3*Δ, *ERG11*Δ, and *ERG3*Δ *+ ERG11*Δ strains showed collateral sensitivity to this drug. Collateral sensitivity is the phenomenon by which a strain acquires increased sensitivity to one drug due to the acquisition of resistance to another drug (here, collateral sensitivity to nitroxoline due to resistance to azoles/polyenes). This phenomenon is well studied in bacteriology and oncology but has only recently been systematically studied in fungi, where collateral sensitivity-based drug cycling was shown to both prevent and actively reduce antifungal drug resistance (27). Besides drug cycling, combination therapy in which collateral sensitive drug pairs are used can be an effective therapeutic improvement. Drugs that cause collateral sensitivity and interact in synergy—when the combined effect of two drugs is greater than their individual effect—would be good candidates for combination therapy (41–44). We did not identify a synergistic interaction between nitroxoline and fluconazole, amphotericin B, or flucytosine, although we also did not detect antagonism. Nevertheless, even if drug pairs are solely additive or even indifferent or antagonistic, they can reduce resistance development, as the type of interactions between antimicrobials—whether synergistic, additive, or antagonistic—does not correlate to evolutionary outcome during combination treatments in bacteria, likely because these interactions can also evolve and collateral sensitivity can play a significant role (43–45). Thus, the absence of synergy does not limit the potential of nitroxoline in combination with antifungals, in complicated UTIs, and we highly recommend further investigations for this purpose.

Nitroxoline is thought to exert its antifungal activity primarily through its ability to chelate metal ions—particularly iron—thereby disrupting metal-dependent processes within the fungal cell. Vincent et al. detected collateral sensitivity toward the iron chelator BPS in *ERG3*Δ *+ ERG11*Δ strains of *C. albicans* (8). Surprisingly, we did not detect collateral or increased sensitivity toward BPS and ciclopirox, known iron chelating antifungal agents, in the constructed mutants and clinical strain. Diiodohydroxyquinoline, an antiparasitic 8-hydroxyquinoline derivative related to nitroxoline, also showed collateral sensitivity in the *ERG11*Δ and *ERG3*Δ *+ ERG11*Δ strains, although the clinical isolate was slightly less susceptible to this compound. In addition, we observed that the supplementation of iron rescues the inhibitory effect of nitroxoline significantly in the wild type strain, while in the *ERG3*Δ *+ ERG11*Δ strain and clinical isolate, this effect was less pronounced. These observations confirm that iron chelation is one of the mechanisms of action of nitroxoline, but they also support the hypothesis that nitroxoline has additional modes of action, beyond iron chelation. Recently, nitroxoline was found to affect membrane integrity in bacteria (30) while in fungi, interactions with alkane 1-monooxygenase and methionine aminopeptidase enzymes were proposed, while no direct effects on cell membrane or cell wall were evidenced (29). Nitroxoline therefore appears to exert a partially distinct mechanism of action compared to other iron chelators tested here. While we cannot yet pinpoint the molecular basis of the mechanism of action or CS phenotype, our data support several plausible, non-mutually exclusive hypotheses. Profound sterol remodeling in *ERG11*- and *ERG3/ERG11*-deficient backgrounds may compromise membrane robustness and transmembrane protein function, potentially altering nitroxoline permeability, uptake, or efflux and thereby increasing intracellular exposure. In parallel, because multiple steps in sterol metabolism are iron-dependent—including the ERG11-associated demethylation step—metal chelation could disproportionately destabilize cells with already perturbed sterol biosynthesis, amplifying physiological fragility and nitroxoline sensitivity. These hypotheses remain to be tested and provide a promising direction for future research.

In summary, this case highlights that even profoundly “unfit” fungal strains can thrive under intensive antifungal therapy when hyper-resistance to multiple drugs is acquired, challenging the traditional premise that severe fitness costs necessarily preclude *in vivo* persistence under treatment. The promising *in vitro* efficacy and collateral sensitivity observed for nitroxoline against these isolates underscore its potential as a viable alternative agent when conventional treatments fail. Taken together, these findings emphasize the dynamic interplay between fitness trade-offs and resistance evolution and point to repurposed therapeutics—like nitroxoline—as essential antifungal agents for recalcitrant MDR fungal infections.

## MATERIALS AND METHODS

### Strains and growth conditions

Strains were stored at −80°C in 20% glycerol and routinely plated on solid YPD (1% yeast extract, 2% bacteriological peptone, 2% dextrose) agar (2%) at 37°C. Unless specified otherwise, cells were grown in MOPS (morpholinopropane sulfonic acid)-buffered (pH 7) RPMI 1640 (Thermo Fisher Scientific) medium with 2% total glucose at 37°C.

### Whole-genome sequencing (WGS)

The clinical *N. glabratus* isolate was isolated from the patient, cultured on Sabouraud dextrose agar and incubated at 37°C for 48 h. Genomic DNA was extracted using the ZymoBIOMICS DNA/RNA Miniprep Kit (Zymo Research, USA), according to the manufacturer’s guidelines. The purified genomic DNA was quantified using a Qubit fluorometer. Library and sample preparation were carried out using the Nextera XT Sample Preparation Kit (Illumina, USA). Sequencing was performed on a MiSeq platform (Illumina Inc., USA) using 2 × 250 bp paired-end sequencing, v2, 500 cycles.

### WGS data analysis

Whole-genome sequencing analysis was performed using the perSVade software (version 1.02.6) (46). To remove adaptors and trim the reads for each sample, we used FastQC (v.0.11.9) (47) and Trimmomatic (v.0.38) (48) with default parameters, facilitated by the trim_reads_and_QC module of perSVade. Alignment of the trimmed reads (with BWA MEM) (v.0.7.17) to the *N. glabratus* ATCC2001 (CBS138) reference genome (version s02-m07-r35), available on the Candida Genome Database, was performed with the align_reads module of perSVade. Genome-wide coverage distributions were visualized using WGSCoveragePlotter, a component of the Jvarkit suite (v. c789c6a41; 10.6084/m9.figshare.1425030; see Fig. S4). After that, the module call_small_variants integrates results of variant calling on the aligned reads (SNPs and small indels) from three different variant callers: BCFtools (v.1.9) (49), GATK HaplotypeCaller (v.4.1.2) (50), and FreeBayes (v.1.3.1) (51).

For variant calling, we used the parameter “–ploidy 1,” under the assumption that the strain has the canonical ploidy. Variants with coverage below 12 and a minimum fraction of reads covering a variant (--min_AF) below 0.9 were filtered out. Moreover, obtained variants were filtered to retain only those that passed the filters of a minimum of two callers. Frameshift variants with coverage of more than 20 that did not initially meet the original minimum allele frequency (min_AF) threshold but were supported by all three callers (lowering the min_AF threshold to 0.85) were included in the final set of variants. This approach mitigates tool-specific biases in indel calling, such as differences in local realignment and breakpoint variability across the used variant callers.

Next, we performed a phylogenetic tree reconstruction for the clinical strain of this study with 420 *N. glabratus* strains (393 of them have a clinical origin) studied in Schikora-Tamarit and Gabaldón (16). Using variant calling data for each strain, we constructed a multi-sample VCF file and generated a pseudo-alignment using the vcf2phylip tool. To avoid the biases introduced by indels, only homozygous SNPs were included in the pseudo-alignment. We then obtained the unrooted tree, using IQ-TREE (v.2.1.2) from this pseudo-alignment using “-m TEST” to use default automatic model selection. Ascertainment bias correction (+ASC) was not included in the model, as the primary objective of the phylogenetic tree reconstruction was not to estimate branch lengths, but to place the strain of interest within the pre-established phylogeny of strains and identify the most likely clade this strain belongs to. Next, we used midpoint rooting to obtain the final tree, which has support values from 1,000 bootstraps.

After determining the clade, all the members of this clade were used to create an artificial background, consisting of SNPs present in more than 20% of the clade members, to filter the variant calling results of the clinical strain and remove SNPs that were identified because of the differences between this strain and the reference genome used. Variants included in the artificial background were filtered out from the high-confidence sets of the clinical strain (with BCFtools v. 1.15.1 [49] function isec). After that, the annotation of final sets of variants was performed with the annotate_small_vars module of Ensembl Variant Effect Predictor v.100.2 (52), incorporated in perSVade.

The filtering criteria for variant calling of the studied strain were selected to align with the established variant calling procedure used in Schikora-Tamarit and Gabaldón (16). This consistency ensures that the clinical strain is consistently and accurately placed within the reconstructed phylogenetic tree.

### Clinical variant data analysis

The co-occurrence of protein-altering variation in both *ERG3* and *ERG11* was analyzed by mining the data set of Schikora-Tamarit and Gabaldón (16). This data set comprises variant calling results for 2,000 genomes of *Candida albicans*, *Candida auris*, *Nakaseomyces glabratus* (*C. glabrata*), *Candida orthopsilosis*, *Candida parapsilosis*, and *Candida tropicalis*.

Protein-altering variants were identified based on Variant Effect Predictor annotations generated in Schikora-Tamarit and Gabaldón (16).

The frequency of each variant was calculated as the proportion of strains with a variant relative to the total number of strains analyzed for each species. Variants in genes of interest were extracted using corresponding gene IDs for *ERG3* and *ERG11* for each species, as annotated in the reference genomes of each species.

Only protein-altering variants, including stop-gained, frameshift, in-frame insertions/deletions, and missense variants with a frequency of less than 0.2 (<20%) were considered in this analysis. This approach aimed to eliminate background genetic variation characteristic of the species or clade, highlighting mutations that could represent recent or species-specific adaptations with potential phenotypic relevance.

### Strain construction

Deletion cassettes for *ERG3* (CAGL0F01793g) and *ERG11* (CAGL0E04334g), consisting of a nourseothricin resistance marker flanked by Flp recombinase recognition sites and 500 bp upstream and downstream homology regions of the target genes, were constructed in the pYC44 vector. The 500 bp upstream and downstream regions of *ERG3* and *ERG11* were amplified from the genomic DNA of the wild-type strain (ATCC2001) with primers designed to include an EciI recognition site at the cassette’s outer ends. Primers are specified in Table S3. The pYC44 vector was digested with XhoI and BamHI, and the promoter and terminator regions were inserted via Gibson Assembly. Correct insertion was verified by PCR using primers listed in Table S3. To generate linear deletion cassettes, the plasmid was digested with EciI.

*N. glabratus* deletion strains for *ERG3* and *ERG11* were generated in the ATCC2001 (CBS138) wild-type background. Overnight cultures in YPD medium were grown at 37°C in a shaking incubator, followed by dilution to OD_600_ 0.4 in 50 mL YPD. Cells were incubated for 3 h at 37°C with shaking, harvested at 3,500 rpm for 5 min, and washed twice with Milli-Q water. The pellet was resuspended in 10 mL of 100 mM LiAc prepared in TE buffer (1×) and incubated at 37°C for 30 min with shaking. DTT (1 M, 250 µL) was added, and cells were incubated for an additional hour. Ice-cold Milli-Q water (40 mL) was added, and cells were harvested (3,500 rpm, 5 min, 4°C), then washed once in ice-cold Milli-Q water and once in 5 mL ice-cold sorbitol (1 M). The final pellet was resuspended in 500 µL ice-cold sorbitol (1 M). For electroporation, 1 µg of linearized deletion cassette was mixed with 40 µL of the prepared cells and transferred into a 2 mm gap electroporation cuvette. Cells were pulsed (1.5 kV, 200 Ω, 25 µF) and recovered in 2 mL YPD at 37°C with shaking for 3 h. Cells were spun down (4,500 rpm, 2 min), resuspended in 100 µL Milli-Q water, and plated on YPD agar supplemented with 200 µg/mL nourseothricin. For *ERG11* deletions, plates were further supplemented with 35 µg/mL ergosterol and 0.0875% (vol/vol) Tween 80 and incubated anaerobically at 37°C, following recommendations from Geber et al. (11). Correct integration of the deletion cassette was confirmed by PCR using primers listed in Table S3.

For *ERG3* deletion strains, marker recycling was achieved via heat-shock with the pLS10 plasmid to express flippase. Overnight cultures in YPD medium were grown at 37°C in a shaking incubator, followed by dilution to OD_600_ 0.4 in 50 mL YPD. Cells were incubated for 3 h at 37°C with shaking, harvested at 3,500 rpm for 5 min, and washed twice with Milli-Q water and once in 1 mL 100 mM LiAc. The pellet was resuspended in 500 µL 100 mM LiAc. For the transformation reaction, 50 µL cells were mixed with 240 µL 50% (wt/vol) PEG, 36 µL 1 M LiAc, 25 µL boiled salmon sperm DNA (2 mg/mL), and 1 µg of pLS10 plasmid in 50 µL Milli-Q water. The mixture was incubated at 30°C for 30 min in a shaking heat block, then heat-shocked at 42°C for 22 min. Cells were recovered in 2 mL YPD at 37°C for 3 h, plated on YPD agar containing 300 µg/mL hygromycin, and incubated at 37°C. Loss of the nourseothricin resistance marker was confirmed by PCR using primers in Table S3. To remove the pLS10 plasmid, cells were plated on non-selective YPD medium and verified by replating on YPD supplemented with 300 µg/mL hygromycin.

### Drug and stress susceptibility testing

Antifungal susceptibility testing was done by the EUCAST reference method (15), Sensititre YeastOne 10, and ETEST (bioMérieux) methods, following manufacturer’s instructions. An adaptation of the EUCAST method was used for drug and stress broth dilution assays (BDA). Briefly, a twofold dilution range of drug was prepared in a total volume of 200 µL RPMI-MOPS (pH 7, 2% glucose, 1% DMSO) medium with approximately 200 cells (based on OD_600_ and serial dilution) in a round-bottom 96-well polystyrene microtiter plate (Greiner). The highest drug concentration was 16 µg/mL for all drugs except anidulafungin (max 8 µg/mL), micafungin (max 1 µg/mL), and flucytosine (max 4 µg/mL). All drugs except fluconazole were dissolved in 100% DMSO, while a final concentration of 1% DMSO was obtained in the final assay. Stressor dilution ranges were 10 mM–0.0391 mM for hydrogen peroxide (H_2_O_2_; Sigma-Aldrich), CFW (Fluorescent Brightener 28, Sigma-Aldrich), and CR (Sigma-Aldrich), 2.5 M–0.0098 M for sodium chloride (NaCl, Sigma-Aldrich), 3.5 mM–0.014 mM for SDS (Sigma-Aldrich), and 500 µM–1.95 µM of BPS (Sigma-Aldrich) in the final concentration of the BDA. Plates were incubated at 37°C for 48 h, and growth was assessed spectrophotometrically (OD_600_) using a Synergy H1 microplate reader (BioTek). Stress susceptibility was assessed using the same protocol.

The growth cut-off for all MIC values from BDA was 50% growth compared to the drug-free control, while in ETEST (bioMérieux), the MIC was indicated by the boundary of the growth inhibition zone on the strip, following the manufacturer’s guidelines.

### Checkerboard assays

For checkerboard assays, the BDA protocol described above was used, but two drugs were serially diluted across two gradients (one vertical for NIT, and one horizontal for AMB, 5FC, or FLU). To evaluate drug interactive effects, the FICI was calculated and indicated in the upper right corner. $FICI=FIC_{drug1}+\;FIC_{drug2}=\frac{MIC_{drug1\;in\;drug2}}{MIC_{drug1}}+\frac{MIC_{drug2\;in\;drug1}}{MIC_{drug2}}$, with MIC_drug1 in drug2_ being the MIC of drug 1 in the presence of the highest concentration of drug 2 where growth is still visible (>50% compared to control without drug). FICI ≤ 0.5 indicates synergy, 0.5 < FICI < 1 indicates an additive effect, 1 ≤ FICI < 4 indicates indifference, and a FICI > 4 indicates antagonism (33). For checkerboard assays in which the MIC of one of the two drugs could not be determined within the tested range, no FICI could be calculated.

### Iron supplementation assays

To assess whether iron supplementation could rescue growth inhibition by nitroxoline or BPS, modified checkerboard assays were performed. As in standard checkerboard assays (see above), the compound of interest was twofold serially diluted across one axis of a 96-well plate. Along the other axis, either iron(III) chloride (FeCl₃) or iron(II) sulfate (FeSO₄) was serially diluted. Iron(III) chloride (FeCl_3_) [iron (III) chloride hexahydrate; Sigma] and iron(II)sulfate (FeSO_4_) [iron(II) sulfate heptahydrate; Merck] were dissolved in Milli-Q water and filter sterilized. The stocks were prepared on the day of the experiment. Plates were incubated at 37°C for 48 h and growth was assessed spectrophotometrically (OD_600_) using a Synergy H1 microplate reader.

### Growth analysis

Cultures were diluted in 200 µL RPMI-MOPS (with 0.2% or 2% glucose) or YPD (2% glucose) to a final cell concentration of 10^6^ cells per well. Growth was monitored at 37°C using spectrophotometric analysis at an optical density of 600 nm (OD_600_) with a Multiskan GO automated plate reader (Thermo Scientific) in flat-bottom 96-well microplates (Greiner), with intermittent (10 min interval) pulsed (1 min medium-strength shaking) shaking and 30 min interval OD_600_ measurements. Growth curves were generated based on two replicate measurements per strain.

### Live/dead cell estimation

Cells were grown on YPD agar medium from a −80°C stock (YPD, 20% glycerol), for 48 h at 37°C. Next, a colony was resuspended in RPMI-1640 MOPS (pH 7) medium with 2% (wt/vol) glucose and processed immediately (t = 0) or after 6 or 24 h incubation at 37°C in a shaking incubator. Cell suspensions were harvested by centrifugation at 3,500 × *g* for 5 min and subsequently washed once with phosphate-buffered saline (PBS). Cell pellets were resuspended in PBS containing 25 ng/mL CFW (Sigma-Aldrich) and 1 µL/mL LIVE/DEAD Fixable Olive stain (Thermo Fisher Scientific). The suspension was incubated for 30 min in the dark at room temperature and washed once with PBS. Cells were visualized on glass slides using an Olympus Fluoview FV1000 inverted epi-fluorescence microscope equipped with a 60× (NA = 1.34, UPLSAPO) objective lens. Fluorescence excitation for CFW was performed using 405 nm light, while the LIVE/DEAD stain was excited using 488 nm light from an argon laser.

Images obtained from confocal microscopy were processed with ImageJ (v.2.16.0). The blue (CFW) and green (LIVE/DEAD) channels were first adjusted to a max threshold of 200–250. Then, the images were despeckled and the binary process watershed was applied to separate adjacent cells. Cell counts were obtained with the “Analyze particles” function with a lower detection limit of 50 pixels as shown in Fig. S2. Total cell counts were determined using the counts from the blue channel (CFW), while dead cells were identified using the cell counts from the green channel (LIVE/DEAD). Two images per strain per time point (45 mm² each) were analyzed. Data analysis, normalization, and visualization were performed using GraphPad Prism v.10.4.2.

### Membrane sterol analysis

Sterols were extracted and analyzed based on Morio et al. (53) with some modifications. Stationary phase cultures were obtained by growing cells in 5 mL RPMI-MOPS (pH 7, 2% glucose) medium in a shaking incubator at 37°C for 48 h. Cells were harvested by centrifugation, washed twice with Milli-Q H_2_O, and a pellet of 20 mg of cells was stored at −80°C. The pellet was resuspended (vortexing for 1 min) in 300 µL saponification medium (12.5 g KOH in 18 mL Milli-Q H_2_O diluted to 50 mL with 98% ethanol), transferred to a capped glass vial, and incubated for 1 h in a shaking water bath at 80°C. Sterols were extracted by adding 100 µL Milli-Q H_2_O and 400 µL hexane, including 1 µL of 5 mg/mL 5-α-cholestane as internal standard (Sigma, 47124), followed by vortexing for 3 min, 20 min phase separation, and careful collection of 350 µL of the top (hexane) layer. A second extraction fraction was collected by adding 600 µL hexane, vortexing for 3 min, 20 min phase separation, and collection of 550 µL of the top (hexane) layer. The two collected hexane fractions were combined and dried using vacuum centrifugation (Automatic Environmental SpeedVac System AES2010) for 30 min at room temperature. Sterol extracts were re-dissolved in 60 µL hexane and derivatized by adding 10 µL of a silylating mixture (Sigma, 85432), short vortexing, and incubation at room temperature for 1 h. Derivatized extracts were shortly centrifuged to precipitate potential debris, and 50 µL of the extract was transferred to a smaller insert glass tube for GC-MS analysis.

The samples were analyzed using a Thermo Scientific gas chromatography-mass spectrometer (Trace 1300–ISQ QD equipped with a TriPlus RSH autosampler and a Restek Rxi-5ms capillary GC column [30 m × 0.25 mm ID]). Helium was used as carrier gas with a flow rate of 1.4 mL/min. Injection was carried out at 250°C in split mode after 1 min and with a ratio of 1:10. The temperature was first held at 50°C for 1 min and then allowed to rise to 260°C at a rate of 50°C/min, followed by a second ramp of 2°C/min until 325°C was reached; that temperature was maintained for 3 min. The mass detector was operated in scan mode (50 to 600 atomic mass units), using electron impact ionization (70 eV). The temperatures of the MS transfer line and detector were 325°C and 250°C, respectively. Sterols were identified by their retention time relative to the internal standard (cholestane) and specific mass spectrometric patterns using Chromeleon 7 (Thermo Scientific). The spectra were matched to GC-MS libraries described in Müller et al. (54) and NIST/EPA/NIH version 2. Analysis was performed by integration over the base ion of each sterol. Abundance was calculated relative to the internal standard, comparing the relative peak areas of the compounds.

Sterol extraction and analysis of each strain were performed in duplicate (technical repeats) on individually cultured strains.

## Data Availability

Whole-genome sequencing data have been deposited as follows: BioProject, PRJNA1258199; BioSample, SAMN48309051 (Nglab_IsolateC [TaxID: 5478]); SRA, SRR33411312.

## SUPPLEMENTAL MATERIAL

Supplemental material mbio.00588-26-s0001.pdf Supplemental figures and tables.

ASM does not own the copyrights to Supplemental Material that may be linked to, or accessed through, an article. The authors have granted ASM a non-exclusive, world-wide license to publish the Supplemental Material files. Please contact the corresponding author directly for reuse.
