# Supplementary material for: Urosepsis by multidrug-resistant Nakaseomyces glabratus with non-functional Erg3 and Erg11—do collateral sensitivity and a unique mode of action make nitroxoline a viable UTI antifungal?
Source: mBio. 2026 May 20;17(6):e00588-26. doi: 10.1128/mbio.00588-26 (PMC13251396; doi:10.1128/mbio.00588-26)
Supplement: Supplemental material — Supplemental figures and tables. [file mbio.00588-26-s0001.pdf]

Supplementary

Figure S1: Formalin-fixed paraffin embedded sections of fibrinpurulent material from TURP show fungal structures in a Grocott-Gomori methenamine silver (GMS) staining (**A**) and inflammatory infiltration of immune cells in a Periodic acid–Schiff (PAS) amylase stain (**B**).

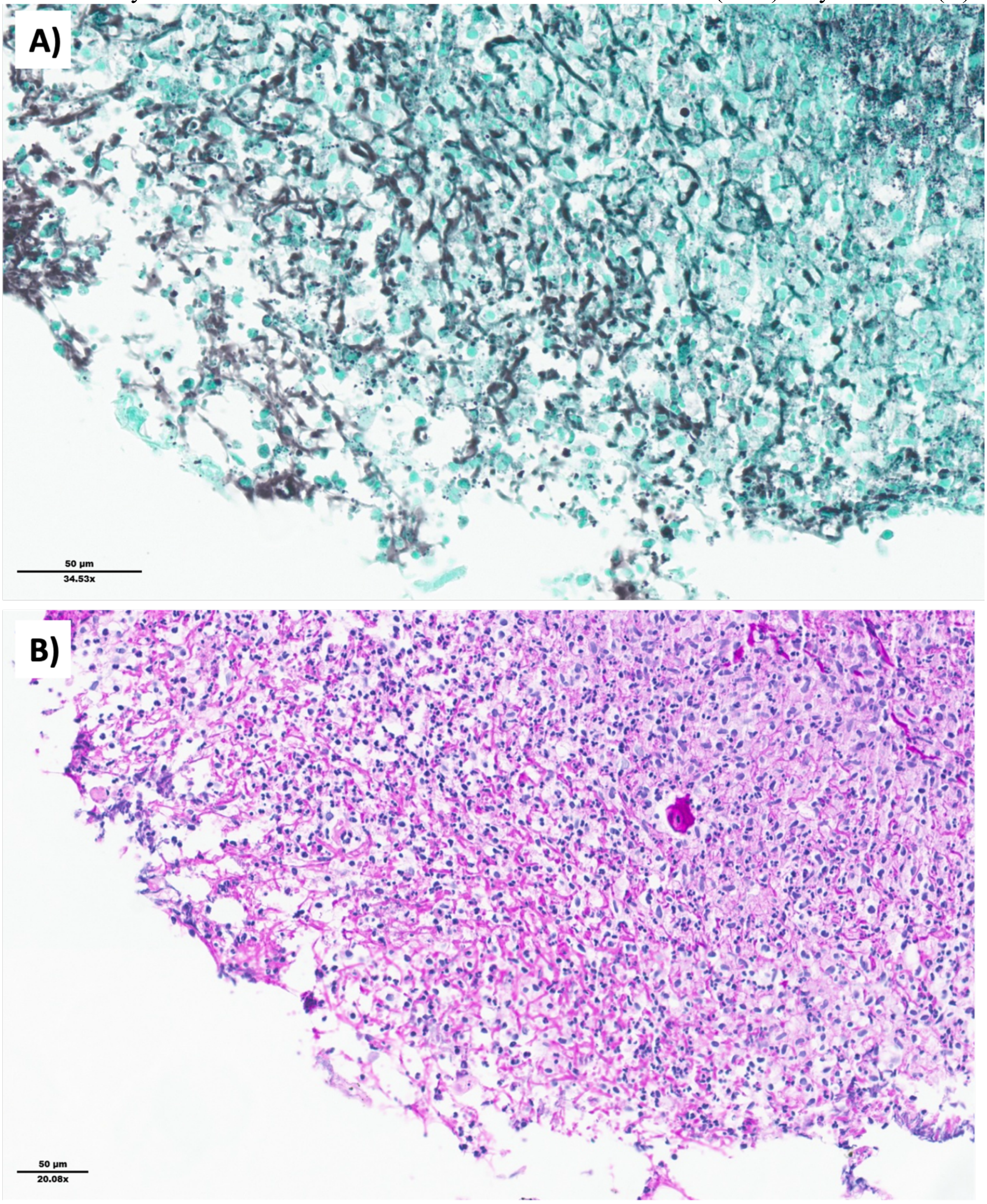

Figure S2: The workflow for quantifying live and dead cells using Differential interference contrast (DIC) confocal fluorescence microscopy. Total cell counts were determined using Calcofluor White (CFW) staining, while dead cells were identified using the LIVE/DEAD™ Fixable Olive (557) Viability Kit (Thermo Fisher Scientific). Images of 45 mm² were analyzed with ImageJ (v2.16.0) by adjusting thresholds and analyzing particles larger than 50 pixels². Outlines of counted particles are shown in the third row. The strain shown is C1 at t=6h, scale bar is 20µm.

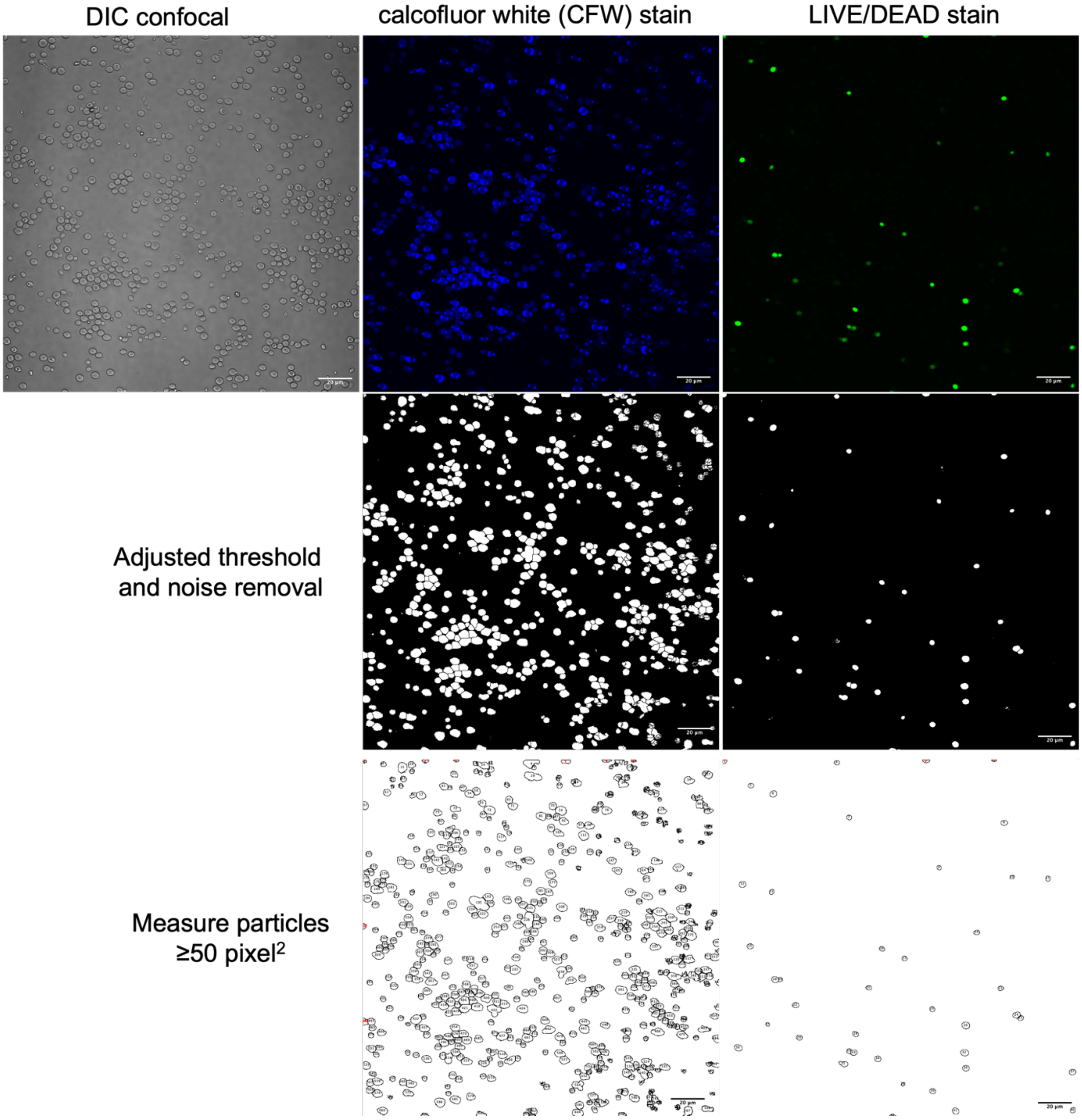

Figure S3: Iron rescue assays to evaluate the bioactivity of BPS against the WT, *ERG3Δ+ERG11Δ* (double deletion, dd) , and clinical (C1) strain in the presence of $FeSO_4$ and $FeCl_3$. BPS and iron were each serially diluted two-fold across a 96-well plate, similarly to a checkerboard assay. Absorbance at 600 nm was measured after 48 h of incubation at 37 °C, and the relative growth was plotted.

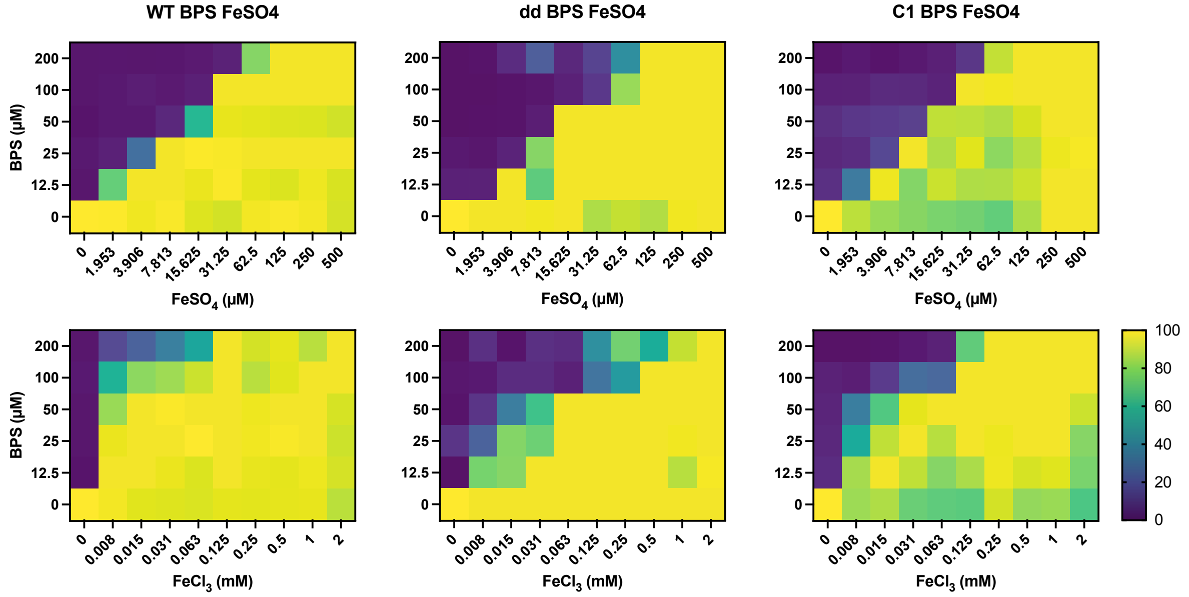

Figure S4: Genome-wide coverage plot of isolate C. Coverage depth (y-axis) is plotted against chromosomal positions (x-axis), with individual chromosomes labelled for clarity. The green and red lines represent the average and median coverage depth, respectively. The peak in coverage in ChrL corresponds to the cluster of rRNA genes, such as RDN25-1 (25S rRNA), RDN58-1 (5.8S rRNA), RDN18-1 (18S rRNA), and RDN5-1 (5S rRNA).

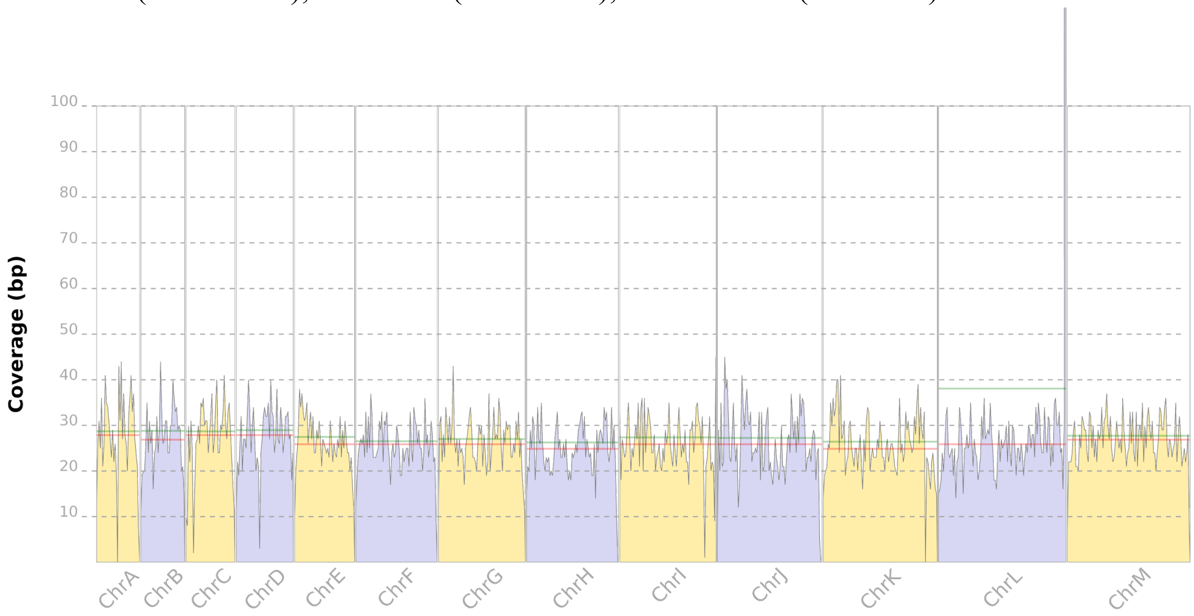

Table S1: List of all protein-altering variants detected in the clinical isolate C. Given are gene IDs from the *N. glabratus* reference genome (ATCC2001/CBS6318), gene aliases or orthologous gene names in *S. cerevisiae* [marked with an asterisk (GENE*)], the mutation's functional consequence, CDS and protein positions, specific amino acid and codon changes, and corresponding BLOSUM62 scores for amino acid substitutions.

Gene ID	Gene	Consequence	CDS position	Protein position	Amino acids	Codons	BLOSUM62
CAGL0C03575g	**AGA1***	missense_variant	1727	576	A/D	gCt/gAt	-2
CAGL0M11770g	**AVL9***	missense_variant	391	131	I/L	Ata/Tta	2
CAGL0H10626g	**AWP13**	missense_variant	2570	857	M/T	aTg/aCg	-1
CAGL0H01419g	**BFR2***	missense_variant	301	101	D/N	Gat/Aat	1
CAGL0K06105g	**BOP2***	missense_variant	268	90	N/D	Aat/Gat	1
CAGL0D03960g	**BRL1***	missense_variant	898	300	Q/E	Cag/Gag	2
CAGL0J09350g	**CDC48**	missense_variant	2273	758	E/A	gAg/gCg	-1
CAGL0J04642g	**DCK1***	missense_variant	4754	1585	S/N	aGt/aAt	1
CAGL0M00902g	**DIF1***	inframe_insertion	350-351	117	*/*IDFS	tga/tgAATCGACTTCTCa	
CAGL0L12782g	**DIG1**	inframe_deletion	368-373	123-125	AVS/A	gCAGTATca/gca	
CAGL0F04521g	**ECM13***	inframe_insertion	769-770	257	D/AFESYDD	gat/gCTTTCGAATCTTACGATGat	
CAGL0A01284g	**EPA10**	missense_variant	2764	922	V/I	Gtt/Att	3
CAGL0J11968g	**EPA15**	missense_variant	1482	494	M/I	atG/atA	1
CAGL0F01793g	**ERG3**	stop_gained	711	237	Y/*	taC/taA	
CAGL0L00671g	**FCY21**	missense_variant	774	258	K/N	aaG/aaT	0
CAGL0M13827g	**FKS3**	missense_variant	2492	831	F/S	tTt/tCt	-2
CAGL0H09064g	**FUR1**	missense_variant	413	138	P/Q	cCa/cAa	-1
CAGL0H06215g	**GAL11A**	missense_variant	824	275	R/H	cGt/cAt	0
CAGL0B02607g	**HEM1**	missense_variant	524	175	L/S	tTg/tCg	-2
CAGL0H08107g	**HMS1***	missense_variant	308	103	P/L	cCa/cTa	-3
CAGL0D04510g	**HOB2***	missense_variant	5135	1712	A/V	gCg/gTg	0
CAGL0M09999g	**IRA1***	missense_variant	1172	391	A/E	gCa/gAa	-1
CAGL0I07909g	**IRA2***	missense_variant	1720	574	I/V	Atc/Gtc	3
CAGL0F06919g	**IRC24***	missense_variant	619	207	T/A	Aca/Gca	0
CAGL0D03322g	**IZH3***	missense_variant	826	276	Q/E	Caa/Gaa	2
CAGL0G06248g	**MAK16***	missense_variant	724	242	D/N	Gat/Aat	1
CAGL0E02035g	**MCH4***	missense_variant	1441	481	P/S	Cct/Tct	-1
CAGL0J04224g	**MDJ1***	missense_variant	836	279	K/R	aAa/aGa	2
CAGL0F07733g	**MLH1***	missense_variant	209	70	P/L	cCt/cTt	-3
CAGL0A01089g	**PBI1***	missense_variant	208	70	P/A	Ccg/Gcg	-1
CAGL0I10340g	**PWP5**	missense_variant	1625	542	S/N	aGc/aAc	1
CAGL0A04741g	**RET2***	missense_variant	812	271	R/H	cGt/cAt	0
CAGL0J11242g	**RHO5***	missense_variant	535	179	Y/H	Tac/Cac	2
CAGL0I03828g	**RPB1**	inframe_deletion	5037-5057	1679-1686	TSPSYSPT/T	acCTCTCCATCGTACTCTCCAACt/act	
CAGL0A03454g	**RSC58***	missense_variant	824	275	G/V	gGa/gTa	-3
CAGL0K12056g	**RSM10***	missense_variant	428	143	T/S	aCc/aGc	1
CAGL0K03773g	**SAS2***	missense_variant	286	96	I/V	Att/Gtt	3
CAGL0F05797g	**SCC2***	missense_variant	238	80	P/T	Cca/Aca	-1
CAGL0G00308g	**SCW4**	inframe_insertion	235-236	79	Q/QQE	caa/cAACAAGaa	
CAGL0M09933g	**SEI1***	missense_variant	173	58	F/Y	tTt/tAt	3
CAGL0F08811g	**SNU71***	missense_variant	1022	341	T/M	aCg/aTg	-1
CAGL0M12254g	**SPC72***	missense_variant	1858	620	L/M	Ttg/Atg	2
CAGL0E01331g	**SWI5**	inframe_insertion	294-295	98-99	-/QSHSQS	-/CAATCGCACTCGCAGTCG	
CAGL0L08778g	**TAF3***	missense_variant	538	180	S/P	Tct/Cct	-1
CAGL0H00572g	**TDA4***	missense_variant	343	115	M/V	Atg/Gtg	1
CAGL0E04972g	**TIP20***	missense_variant	781	261	Y/N	Tat/Aat	-2
CAGL0F01463g	**TIR1**	inframe_deletion	364-381	122-127	SAAPSS/-	TCTGCCGCTCCATCTTCT/-	
CAGL0F00495g	**TOR1***	missense_variant	3532	1178	K/E	Aag/Gag	1
CAGL0C00605g	**UF***	missense_variant	274	92	N/Y	Aat/Tat	-2
CAGL0C00968g	**UF***	missense_variant	2237	746	G/A	gGt/gCt	0
CAGL0C01133g	**UF***	missense_variant	140	47	T/N	aCt/aAt	0
CAGL0C01133g	**UF***	missense_variant	221	74	T/R	aCa/aGa	-1
CAGL0C01133g	**UF***	missense_variant	2440	814	S/A	Tct/Gct	1
CAGL0C01133g	**UF***	missense_variant	224	75	G/V	gGa/gTa	-3
CAGL0C01133g	**UF***	missense_variant	2029	677	F/V	Ttt/Gtt	-1
CAGL0C01133g	**UF***	missense_variant	220	74	T/A	Aca/Gca	0
CAGL0C01133g	**UF***	missense_variant	146	49	G/A	gGt/gCt	0
CAGL0C01133g	**UF***	missense_variant	215	72	G/D	gGt/gAt	-1
CAGL0C01133g	**UF***	missense_variant	212	71	A/G	gCt/gGt	0
CAGL0F04939g	**UF***	missense_variant	457	153	L/V	Cta/Gta	1
CAGL0G01738g	**UF***	missense_variant	10	4	T/A	Aca/Gca	0
CAGL0M01870g	**UF***	missense_variant	367	123	E/K	Gaa/Aaa	1
CAGL0M01914g	**UF***	missense_variant	2000	667	N/S	aAc/aGc	1
CAGL0M04895g	**UF***	missense_variant	1944	648	R/S	agG/agT	-1
CAGL0L08646g	**ULP1***	missense_variant	769	257	P/S	Ccg/Tcg	-1
CAGL0H01683g	**URC2***	missense_variant	347	116	I/N	aTt/aAt	-3

CAGL0C00737g	**UTP13***	missense_variant	565	189	E/K	Gag/Aag	1
CAGL0K05379g	**VMA13**	missense_variant	959	320	C/Y	tGc/tAc	-2
CAGL0C01771g	**VVS1***	missense_variant	1036	346	A/T	Gca/Aca	0
CAGL0I08305g	**YAT2***	missense_variant	2793	931	D/E	gaC/gaG	2
CAGL0E00517g	**YCR087 C-A***	inframe_insertion	199-200	67	K/KKQNQ	aag/aAGAAACAGAATCa g	
CAGL0I01144g	**YPT35***	missense_variant	121	41	R/S	Cgt/Agt	-1
CAGL0L04576g	**YRM1***	inframe_insertion	2238-2239	746-747	-/GTSQGTSQ	- /GGGACCAGCCAGGGG ACCAGCCAG	
CAGL0C05467g	**MGF1***	frameshift_variant, inframe_insertion	60-61	20-21	-/HRX	- /CACAG ACA	
CAGL0E04334g	**ERG11**	frameshift_variant	1543	515	L/X	Cta/ta	
CAGL0F03641g	**UF***	frameshift_variant, inframe_insertion	72-73	24-25	-/NCX	- /AATTG TGC	
CAGL0H00110g	**UF***	frameshift_variant	491-498	164-166	SHS/X	tCCCAT AGT/t	
CAGL0M03333g	**EDC3***	frameshift_variant	1877-1878	626	A/AX	gca/gcTa	

Table S2: List of all samples containing combined *ERG3* and *ERG11* variants with frequency < 0.2. The table includes the SRR ID for each sample, species name, detected variants in ERG3 and ERG11, and corresponding BioProject and BioSample IDs. Additionally, the isolate type is provided as referenced in Schikora-Tamarit & Gabaldón (16).

**Sample**	**Species**	***ERG3***	***ERG11***	**BioProject**	**BioSample**	**Type**
ERR2708451	*C.albicans*	A26T;A351V;A353T	D153E	PRJEB27862	SAMEA4799805	environmental
ERR2708453	*C. albicans*	A26T;A351V;A353T	D153E	PRJEB27862	SAMEA4799805	environmental
ERR2708457	*C. albicans*	A351V;A353T	E266D;V488I	PRJEB27862	SAMEA4799806	environmental
ERR2708458	*C. albicans*	A351V;A353T	E266D;V488I	PRJEB27862	SAMEA4799806	environmental
ERR2708459	*C. albicans*	A351V;A353T	E266D;V488I	PRJEB27862	SAMEA4799806	environmental
ERR2708460	*C. albicans*	A351V;A353T	E266D;V488I	PRJEB27862	SAMEA4799806	environmental
SRR1159247	*C. albicans*	A351V	V437I	PRJNA345600	SAMN02567302	clinical
SRR1159305	*C. albicans*	A351V	V437I	PRJNA345600	SAMN02567303	clinical
SRR1554297	*C. albicans*	A351V	Y132F;V437I	PRJNA257929	SAMN02980805	clinical
SRR1810521	*C. albicans*	A351V;A353T	E266D;V488I	PRJNA73979	SAMN03164130	clinical
SRR1811019	*C. albicans*	A351V;A353T	E266D;V488I	PRJNA73979	SAMN03164130	clinical
SRR2088861	*C. albicans*	A351V;A353T	E266D;V488I	PRJNA271803	SAMN03277227	clinical
SRR392814	*C. albicans*	T311I	V437I	PRJNA75219	SAMN01048007	clinical
SRR393529	*C. albicans*	A351V	E266D;V437I	PRJNA75247	SAMN01048015	clinical
SRR393530	*C. albicans*	A351V;A353T	E266D;V488I	PRJNA75237	SAMN01048012	clinical
SRR5133893	*C. albicans*	A351V	V437I	PRJNA345600	SAMN06173389	clinical
SRR5133895	*C. albicans*	A351V	E266D;V437I	PRJNA345600	SAMN06173391	inmouse_evol_clone
SRR5133898	*C. albicans*	A351V	E266D;V437I	PRJNA345600	SAMN06173386	clinical
SRR5133903	*C. albicans*	A351V	V437I	PRJNA345600	SAMN06173388	clinical
SRR530262	*C. albicans*	T311I	V437I	PRJNA75219	SAMN01048007	clinical
SRR538772	*C. albicans*	A351V	E266D	PRJNA165033	SAMN00974110	clinical
SRR538782	*C. albicans*	A351V	E266D;S405F	PRJNA165029	SAMN00974108	clinical
SRR540283	*C. albicans*	A351V	V437I	PRJNA75227	SAMN00767978	clinical
SRR543721	*C. albicans*	A351V;A353T	E266D;V488I	PRJNA75237	SAMN01048012	clinical
SRR543724	*C. albicans*	A351V	E266D;V437I	PRJNA75247	SAMN01048015	clinical
SRR543726	*C. albicans*	A351V	E266D;V437I	PRJNA75239	SAMN01048013	clinical
SRR629744	*C. albicans*	A351V;A353T	V437I	PRJNA165035	SAMN00974111	clinical
SRR641726	*C. albicans*	A351V	E266D	PRJNA165033	SAMN00974110	clinical
SRR641728	*C. albicans*	A351V	E266D;S405F	PRJNA165029	SAMN00974108	clinical
SRR646260	*C. albicans*	A351V;A353T	V437I	PRJNA165035	SAMN00974111	clinical
SRR647104	*C. albicans*	A351V	E266D	PRJNA165033	SAMN00974110	clinical
SRR647109	*C. albicans*	A351V	E266D;S405F	PRJNA165029	SAMN00974108	clinical
SRR6669857	*C. albicans*	A351V;A353T	E266D;V488I	PRJNA432884	SAMN08465161	clinical

SRR6669865	*C. albicans*	A351V	E266D;V437I	PRJNA432884	SAMN08465241	clinical
SRR6669883	*C. albicans*	A351V	E266D;V437I	PRJNA432884	SAMN08465216	clinical
SRR6669893	*C. albicans*	A351V;A353T	E266D;V488I	PRJNA432884	SAMN08465283	clinical
SRR6669897	*C. albicans*	A351V	E266D	PRJNA432884	SAMN08465227	clinical
SRR6669905	*C. albicans*	A351V	V437I	PRJNA432884	SAMN08465280	clinical
SRR6669908	*C. albicans*	A351V;A353T	E266D;S442F;V488I	PRJNA432884	SAMN08465273	clinical
SRR6669914	*C. albicans*	A351V	F145I;V437I	PRJNA432884	SAMN08465275	clinical
SRR6669917	*C. albicans*	A351V	E266D;V437I	PRJNA432884	SAMN08465194	clinical
SRR6669918	*C. albicans*	A351V	E266D;V437I	PRJNA432884	SAMN08465193	clinical
SRR6669921	*C. albicans*	A351V;A353T	E266D;V488I	PRJNA432884	SAMN08465198	clinical
SRR6669926	*C. albicans*	A351V	E266D;V488I	PRJNA432884	SAMN08465267	clinical
SRR6669935	*C. albicans*	A351V	E266D;I483V	PRJNA432884	SAMN08465270	clinical
SRR6669936	*C. albicans*	A351V;A353T	E266D;V488I	PRJNA432884	SAMN08465203	clinical
SRR6669958	*C. albicans*	A351V;A353T	E266D;V488I	PRJNA432884	SAMN08465253	clinical
SRR6669963	*C. albicans*	A351V;A353T	E266D;V488I	PRJNA432884	SAMN08465177	clinical
SRR6669967	*C. albicans*	A351V;A353T	E266D;V488I	PRJNA432884	SAMN08465173	clinical
SRR6669969	*C. albicans*	H28Y	E266D;V488I	PRJNA432884	SAMN08465171	clinical
SRR6669977	*C. albicans*	A351V;A353T	E266D;V488I	PRJNA432884	SAMN08465325	clinical
SRR6669978	*C. albicans*	A351V;A353T	E266D;V488I	PRJNA432884	SAMN08465326	clinical
SRR6669988	*C. albicans*	A351V	E266D;V437I	PRJNA432884	SAMN08465185	clinical
SRR6669989	*C. albicans*	A351V;A353T	E266D;V437I	PRJNA432884	SAMN08465186	clinical
SRR6669992	*C. albicans*	A351V	V437I	PRJNA432884	SAMN08465189	clinical
SRR6669993	*C. albicans*	A351V;A353T	E266D;V488I	PRJNA432884	SAMN08465190	clinical
SRR6669994	*C. albicans*	H28Y	E266D;V488I	PRJNA432884	SAMN08465320	clinical
SRR6670002	*C. albicans*	A351V	V437I	PRJNA432884	SAMN08465312	clinical
SRR6670004	*C. albicans*	H28Y	E266D;V488I	PRJNA432884	SAMN08465157	clinical
SRR6670013	*C. albicans*	A351V	V437I	PRJNA432884	SAMN08465160	clinical
SRR6670014	*C. albicans*	A351V;A353T	E266D;V488I	PRJNA432884	SAMN08465236	clinical
SRR6670017	*C. albicans*	D14N	E336G	PRJNA432884	SAMN08465310	clinical
SRR6670018	*C. albicans*	H28Y	E266D;V437I	PRJNA432884	SAMN08465232	clinical
SRR7801916	*C. albicans*	A351V	E266D;V437I	PRJNA489773	SAMN09882461	clinical
SRR7801918	*C. albicans*	A351V	E266D	PRJNA489773	SAMN09882469	clinical
SRR7801925	*C. albicans*	A351V	E266D	PRJNA489773	SAMN09882470	clinical
SRR8324560	*C. albicans*	A351V	E266D;V437I	PRJNA510147	SAMN10598544	in_vitro
SRR8324565	*C. albicans*	A351V	V437I	PRJNA510147	SAMN10598547	in_vitro
SRR8324566	*C. albicans*	A351V;A353T	E266D;V488I	PRJNA510147	SAMN10598546	in_vitro
SRR845177	*C. albicans*	A351V	E266D	PRJNA165033	SAMN00974110	clinical
SRR845178	*C. albicans*	A351V	E266D	PRJNA165033	SAMN00974110	clinical
SRR845180	*C. albicans*	A351V;A353T	V437I	PRJNA165035	SAMN00974111	clinical
SRR845262	*C. albicans*	A351V	E266D;S405F	PRJNA165029	SAMN00974108	clinical
SRR10461173	*C. auris*	P151S	Y132F	PRJNA595978	SAMN13294183	clinical
SRR3547474	*C. orthopsilosis*	I105T	Y13C	PRJNA322245	SAMN05149982	clinical
SRR12823704	*C. tropicalis*	K15R;A48T;T66A;S70T;V74I;T144S;M148I;M148V;Y209F;I360V	I25T;I25V;Y79H;Y221F;R245K;K344T;V362I;N364D;E428D;D431A	PRJNA604451	SAMN16435814	environmental
SRR12823705	*C. tropicalis*	K15R;A48T;T66A;S70T;V74I;T144S;M148I;M148V;Y209F;I360V	I25T;I25V;Y79H;Y221F;R245K;K344T;V362I;N364D;E428D;D431A	PRJNA604451	SAMN16435813	environmental
SRR12823706	*C. tropicalis*	K15R;A48T;T66A;S70T;V74I;T144S;M148V;Y209F;V389I	I25T;I25V;Y79H;Y221F;K344N;K344T;V362I;E428D;D431A	PRJNA604451	SAMN16435812	environmental
SRR12823707	*C. tropicalis*	K15R;A48T;T66A;S70T;V74I;T144S;M148V;Y209F;V3	I25T;I25V;Y79H;Y221F;K344N;K344T;V362I;E4	PRJNA604451	SAMN16435811	environmental

		89I	28D;D431A			
SRR12823743	*C. tropicalis*	K15R;A48T;T66A;S70T;V74I;T144S;M148V;Y209F;V389I	I25T;I25V;Y79H;Y221F;V362I;E428D;D431A	PRJNA604451	SAMN16435778	environmental
SRR13132457	*C. tropicalis*	K15R;A48T;T66A;S70T;V74I;T144S;M148V;Y209F;V389I	I25T;I25V;Y79H;Y221F;R245K;K344N;K344T;V362I;E428D;D431A	PRJNA677456	SAMN16773549	clinical

Table S3: oligonucleotides used in this study

Name	Purpose	Sequence (5'-3')
ERG11_FW_PROM_pYC44	Amplify *ERG11* promoter region to insert in pYC44	CGGCCGCTCTAGAACTAGTGGGCGGAAGGAGTACAAGGGTACAAAG
ERG11_RV_PROM_pYC44	Amplify *ERG11* promoter region to insert in pYC44	TAGAAAGTATAGGAACTTCGGTTATTGTAGTTTTTTTTGATTTATGTG
ERG11_FW_TERM_pYC44	Amplify *ERG11* terminator region to insert in pYC44	AGAGAATAGGAACTTCGTCCATGCTTTCAAAATATTAAACATTTTC
ERG11_RV_TERM_pYC44	Amplify *ERG11* terminator region to insert in pYC44	AGCTGGTACCGGGCCCCCCCTCCGCCTGTTTCTTTAGGCTTACC
ERG3_FW_PROM_pYC44	Amplify *ERG3* promoter region to insert in pYC44	CGGCCGCTCTAGAACTAGTGGGCGGAATAAGAATGCAAGTGTACAAG
ERG3_RV_PROM_pYC44	Amplify *ERG3* promoter region to insert in pYC44	TAGAAAGTATAGGAACTTCGTATTTGTTTTTGTATAAAAGCTTAGTG
ERG3_FW_TERM_pYC44	Amplify *ERG3* terminator region to insert in pYC44	AGAGAATAGGAACTTCGTCCACAGGAAACACCGGTGTC
ERG3_RV_TERM_pYC44	Amplify *ERG3* terminator region to insert in pYC44	AGCTGGTACCGGGCCCCCCCTCCGCCAGATCAAAGGTTGGTAAGCC
pYC44_PROM_FW_ CHECK	Check insertion terminator in pYC44	TTCGCTATTACGCCAGCTG
NAT_FW_CHECK	Check insertion terminator in pYC44/ Check integration deletion cassette	CATCATCTGCCCAGATGCGAAG
NAT_REV_CHECK	Check insertion promoter in pYC44/ Check integration deletion cassette	CGTCAAGACTGTCAAGGAGGG
pYC44_TERM_REV_CHECK	Check insertion promoter in pYC44	GCGAGTCAGTGAGCGAGGA
ERG11_FW_CHECK	Check integration deletion cassette	CCATACTACCATCGCCATTC
ERG11_RV_CHECK	Check integration deletion cassette	GTGAGATGATCTGTAGTATGACAGC
ERG3_FW_CHECK	Check integration deletion cassette / Check removal *NAT* marker	AAAAAGTGTATGCAGTGTGAG
ERG3_RV_CHECK	Check integration deletion cassette / Check removal *NAT* marker	GTAGCGTGTGAACTGGGTAT
